# Unravelling the Complexity of Amyloid Peptide Core Interfaces

**DOI:** 10.1021/acs.jcim.4c01479

**Published:** 2024-10-30

**Authors:** Máté Sulyok-Eiler, Veronika Harmat, András Perczel

**Affiliations:** † Medicinal Chemistry Research Group, HUN-REN Research Centre for Natural Sciences, Magyar Tudósok Körútja 2, H-1117 Budapest, Hungary; ‡ Laboratory of Structural Chemistry and Biology, Institute of Chemistry, ELTE Eötvös Loránd University, Pázmány P. stny. 1/A, H-1117 Budapest, Hungary; § Hevesy György PhD School of Chemistry, Institute of Chemistry, Eötvös Loránd University, Pázmány P. stny. 1/A, H-1117 Budapest, Hungary; ∥ HUN-REN-ELTE Protein Modeling Research Group, Hungarian Research Network, Pázmány P. stny. 1/A, H-1117 Budapest, Hungary

## Abstract

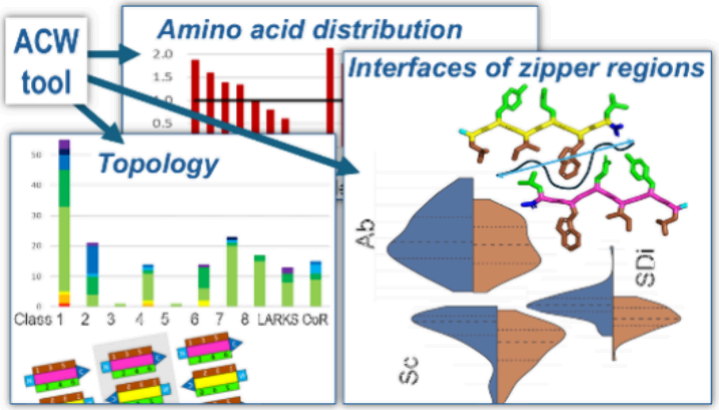

Amyloids, large intermolecular sandwiched β-sheet structures, underlie several protein misfolding diseases but have also been shown to have functional roles and can be a basis for designing smart and responsive nanomaterials. Short segments of proteins, called aggregation-prone regions (APRs), have been identified that nucleate amyloid formation. Here we present the database of 173 APR crystal structures currently available in the PDB, and a tool, ACW, for analyzing their topologies and the 267 inter-β-sheet interfaces of zipper regions assigned in these structures. We defined a new descriptor of zipper interfaces, the surface detail index (SDi), which quantifies the intertwining between the side chains of both β-sheets of the zipper, an important factor for the molecular recognition and self-assembly of these mesostructures. This allowed a comparative analysis of the zipper interfaces and identification of 6 clusters with different intertwining, steric fit, and size characteristics using three complementary descriptors, SDi, shape complementarity, and buried surface area. 60% of the APR structures are formed by parallel β-sheets, of which 52% belong to the topological class 1. This could be explained by the better fit and a deeper entanglement of the zipper regions of the parallel structures than of the antiparallel structures, as the analysis showed that both their shape complementarity (0.79 vs 0.70) and SDi (1.53 vs 1.32) were higher. The higher abundance of certain residues (Asn and Gln in parallel and Leu and Ala in antiparallel β-sheets) can be explained by their ability to form different ladder-like secondary interaction patterns within β-sheets. Analogous to the hierarchy of protein structure, we interpreted the primary, secondary, tertiary, and quaternary structure levels of APRs revealing different characteristics of the zipper regions for both parallel and antiparallel β-sheet structures, which may provide clues to the structural conditions of amyloid core formation and the rational design of amyloid polymorphs.

## 1 Introduction

Amyloids are protein aggregates with a fibrillar morphology that are found as deposits in various organs and tissues. Amyloids have been associated with a wide variety of diseases including Parkinson’s disease, Alzheimer’s disease, type II diabetes, and prion diseases.^1−3^ More recently, functional amyloids such as bacterial curli fibrils and peptide hormone storage have been described.^4,5^ On the other hand amyloid fibrils have opened a new field of research in materials science due to their unique stability, self-recognition, and self-association as structural motifs.^6^ The common structural element of amyloids is their quasi-infinitely long β-sheets forming the core of the fibril which was revealed by the early structural studies as a characteristic cross-β X-ray diffraction pattern.^7^ The first structures of the full-length amyloid fibrils of amyloid β were solved by solution-state NMR in 2005 confirming the stacked β-sheet structure.^8^ Solution state and solid-state NMR^9^ were the only methods to obtain structural information on amyloid fibrils until the first cryo-EM structure of dimer tau protein fibrils was published.^10^ The rapid development of this technique has also led to a major breakthrough, with more than 400 amyloid structures of full-length proteins^11,12^ now deposited in the Protein Data Bank (PDB).^13^ Although the common secondary structural element is the parallel β-sheet (in almost all known structures), the amyloid polypeptide structures usually contain segments of β-sheet secondary structure separated by kinks so that they can exclude water and form interdigitated interfaces between the β-sheet segments, also called dry zippers. The variability of both the kinked conformers and the matching segments within the dry interfaces leads to different amyloid folds, resulting in multiple polymorphic structures of the same amyloidogenic sequence.^11^ Thus, an amyloid structure is the H-bonded ensemble of kinked β-strands forming a curly β-sheet that, if long enough, can even be folded back on itself.

**Figure 1:**
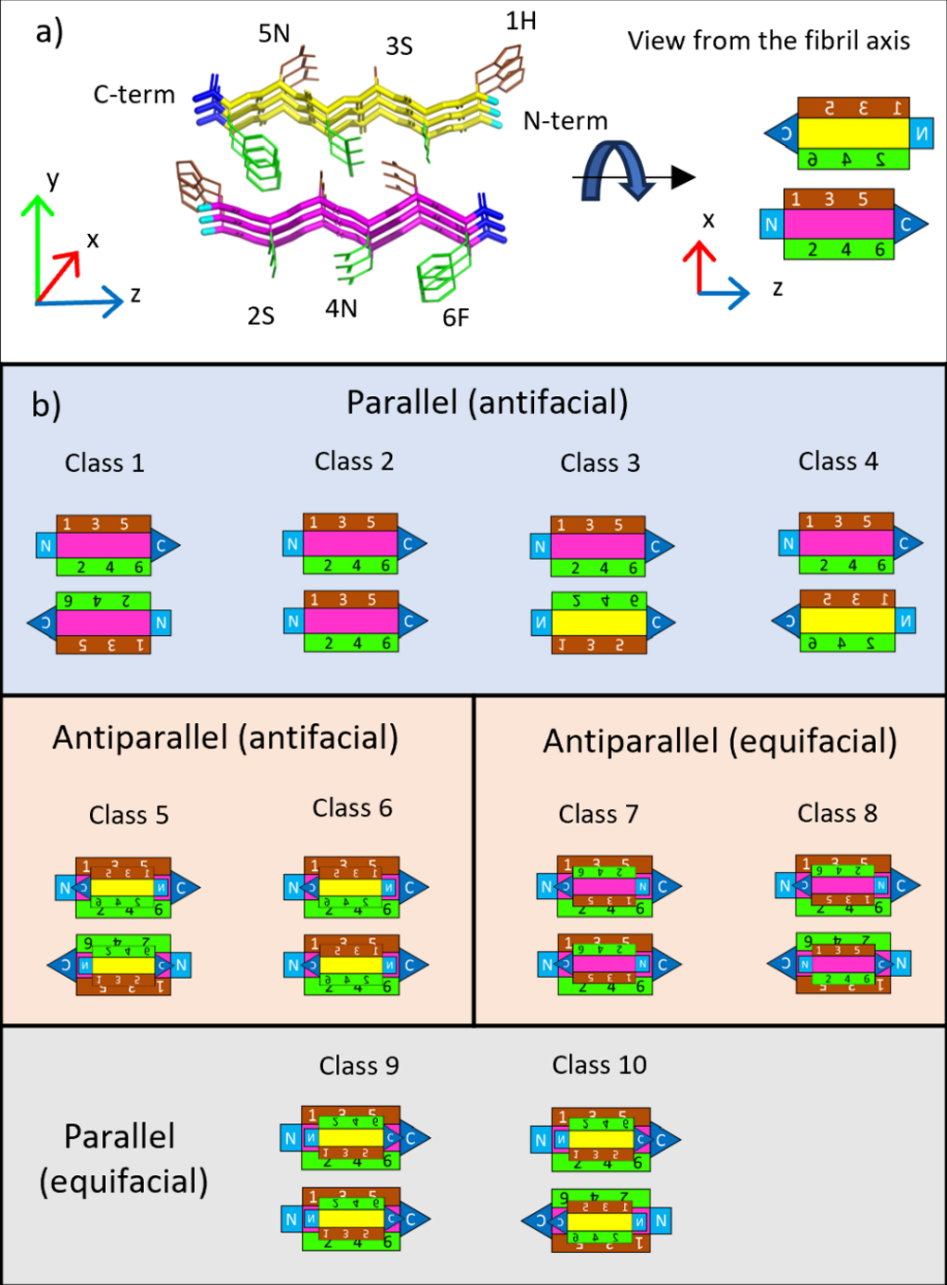
Topological classes of amyloid oligopeptide structures. (a) An example is shown with definition of principal axes, color coding, and schematic “rocket shape” representation of a hexapeptide (right). (PDB ID: 3FPO(36)). The spine of the amyloid contains an “infinite” β-sheet secondary structure element along the *y*-axis. There are interactions between the side chains, forming a tight packing (*x*-direction). Backbones are colored magenta or yellow for the oppositely oriented H-bonds (up–down). For the schematic rocket representation, the β-sheet is viewed from the axis of the fibril. (b) The topologies of the 10 major topology classes^27^ are shown. (The repeating unit is a pair of β-strands for classes 5–10, shown as stacked rocket shapes.) Note that structures belonging to classes 1, 3, and 5 have two different interfaces, only one of which is shown here.

Aggregation-prone regions or APRs are used to model β-sheet formation, which are shorter segments of longer amyloidogenic proteins that are likely to drive amyloid formation.^14−16^ To model β-sheet formation, APRs are used: 5–10 residue-long oligopeptides, of which hexapeptides are the most studied. To date, several APR databases and predictors have been developed based on short (mostly hexapeptide) sequences. The WALTZ-DB^17^ database is a widely used reference, containing only hexapeptide sequences, and their experimentally determined amyloidogenicity (Th-T binding, TEM, crystallography -with reference to experimental structures) together with structural models generated based on experimental target structures. Other databases, such as CPAD 2.0,^18^ contain experimentally validated data, often combined from different sources. Some of the predictors of amyloidogenicity of oligopeptides or protein segments can be based on sequence-based properties (e.g., Tango,^19^ Budapest Amyloid Predictor^20^); or can use amyloid oligopeptide structures from the PDB as templates to generate structures: zipperdb^21^; or can use both approaches (CORDAX server^22^). Prediction of amyloidogenicity can also be based on testing the ability to fold to theoretical structural elements (e.g., to the β-arch structure in the case of ArchCandy^23^). On the other hand, experimentally determined full-length amyloid structures are collected in databases that also provide tools for analyzing the role of individual residues in the structure (Amyloid Atlas,^11^ StAmP^12^). However, to our knowledge, a collection and comparative analysis of all experimentally determined structures of APRs has not yet been published. 3D structures of amyloid oligopeptides were first solved for the yeast protein Sup35 APRs.^24^ Soon, 11 more were added, and a classification system was created^25^ distinguishing different topological classes, based on the parallel/antiparallel organization of the β-sheets and their orientations with respect to the contacting β-sheets within the crystal structure (Figure 1). Examples for classes 3, 5, and 6 were missing at that time, but such structures were published later with the last missing topological element, the class 3 structure published in 2024.^26^ To date more than 200 APR structures have been deposited in the PDB database.^13^

In parallel β-sheets, the two faces of the β-sheet are different (antifacial), so two β-sheets are paired with the same or different sides and facing in the same, or the opposite direction; these four combinations form the basis of topological classes 1–4. In antiparallel β-sheets, the two faces can consist of the same (equifacial) or different (antifacial) sets of residues and can be combined in two different ways to form zipper regions (classes 5–8). Equifacial arrangement is also possible in parallel structures,^27^ giving rise to classes 9 and 10, where the alternating orientation of the β-strands creates an equifacial side, but no such structure has yet been published. Many outliers were also found over time: the two most common groups were named out-of-register (OoR),^28,29^ and low-complexity aromatic-rich kinked segments (LARKS).^30^ In the former one, the H-bonds between the β-strands are out of register, and consequently, the β-strands are not perpendicular to the axis of the β-sheets. In LARKS(s), the main structural feature is the highly kinked backbone fold, which often contains fewer H-bonds.

The zipper-like interface formed by the complementary side chains of the two β-sheets can show different degrees of interdigitation.^31^ It can also have inclusions of solvent molecules or ions, which is the basis of the distinction between dry and wet interfaces. To characterize the interfaces between β**-**sheets, two descriptors are widely used: shape complementarity^32^ (Sc) measures the steric fit of the facing molecular surfaces of the two β**-**sheets. The buried area or solvent-excluded area^33^ (Ab) measures the loss of solvent-accessible area within the interface. However, the use of these descriptors has been ambiguous because they depend on the size of the molecular subset for which they are calculated, which becomes particularly problematic for the solvent-excluded area. For example, for the exact same structure of 2OMM, Ab was first calculated^25^ as 157 Å^2^ and then reported as 542 Å^2^ in a subsequent article.^34^ The large differences come from whether to include or not the Ab between β-strands of the same β-sheet, and to calculate the Ab between a β-sheet and a β-strand or between two β-sheets.

Here, we present the first comprehensive study of the amyloid core structures. Analogous to the structural hierarchy of proteins, we analyze their primary (sequence, amino acid distribution), secondary (backbone conformation), tertiary (topological classes, characterizing the zippers), and quaternary (3D network of wet and dry interfaces within the crystal) structural properties. We revise the commonly used descriptors using a unified method for selecting structural regions of interest and define a new descriptor to characterize the intertwining of the interfaces. The zippers can be visualized and analyzed, both for database members and new structures, as well as using ACW, a Pymol^35^ plugin, a novel tool for automatic analysis of new amyloid crystal structures using topological features and structural descriptors.

## 2 Results and Discussion

216 amyloid core structures were retrieved from the PDB, of which 173 were suitable for study (Tables S1 and S2), with lengths of 3–11 residues, among which the hexapeptides are the most common, a total of 99 unique structures. The database contains 35 new hexapeptide sequences not found in the WALTZ database^17^ and 5 structures with sequences previously assigned as nonamyloidogenic. The 173 suitable structures contain 267 zipper-like interfaces (Table S3). We report our results using the four-level structural hierarchy of proteins: primary, secondary, tertiary, and quaternary structures interpreted for amyloids. Sequences (primary structures) of APRs favor certain topological arrangements (topological classes) of the forming amyloid-like mesostructure via networks of stabilizing interactions, resulting in various, often polymorphic structures. The interactions and complementarities observed between the interacting faces of the β-sheets are essential to understanding the molecular packing at the level of both the tertiary and quaternary structures.

### 2.1 Primary Structure: Analysis and Amino Acid Distribution

**Figure 2:**
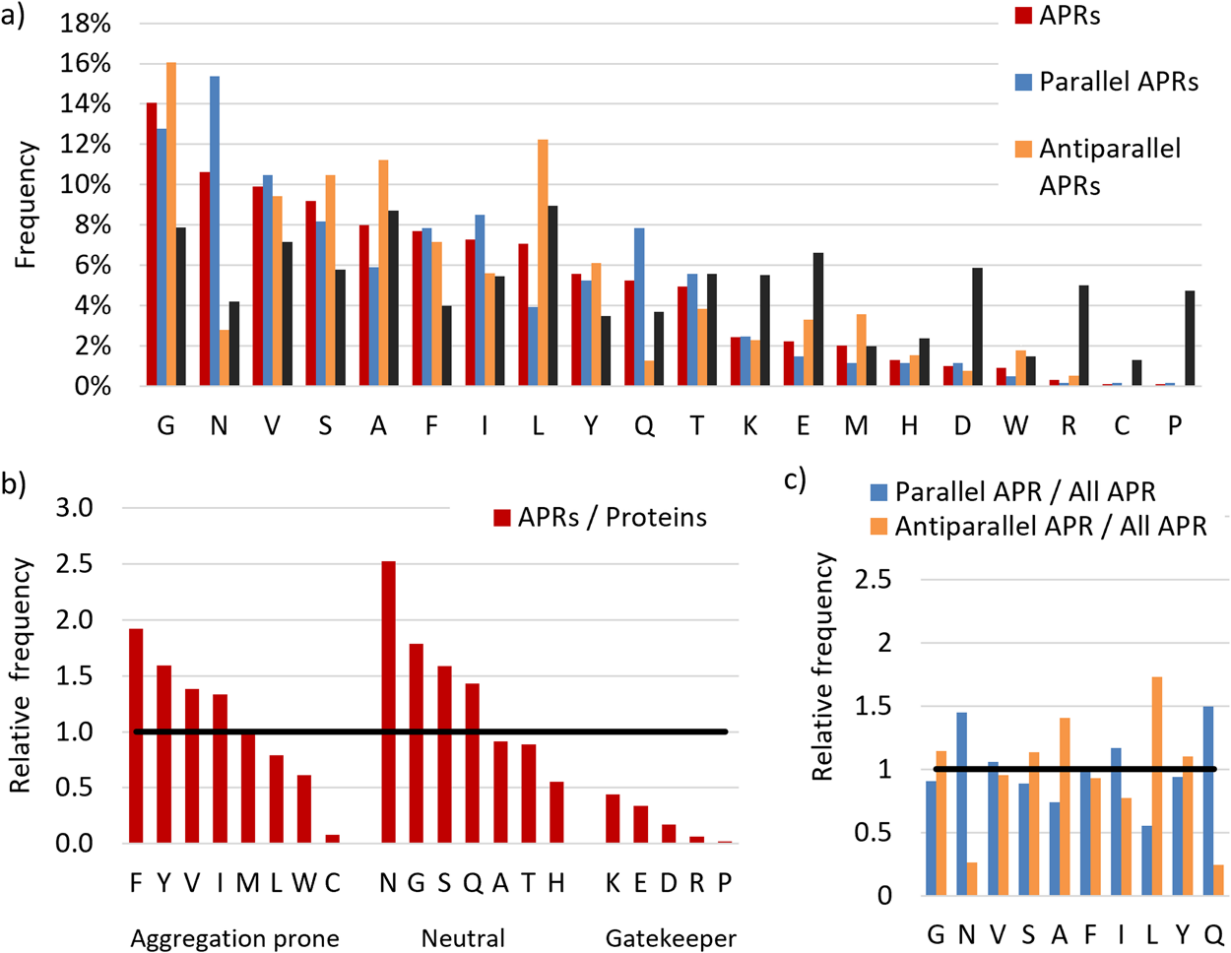
Analysis of the amino acid composition of APR oligopeptide structures. (a) Comparison of the residue composition within the amyloid oligopeptide structures (APRs, red, blue and orange) and that of protein structures (representative selection)^43^ (black). Amino acids are shown in the descending order of their total occurrence in APRs. (b) Comparison of relative occurrence of residues in amyloid core structures to that in protein structures. Amino acids are grouped according to their aggregation propensity (aggregation prone, neutral and gatekeeper, data from ref (38)). (c) Comparison of the relative propensity of residues for parallel (blue) and antiparallel (orange) APRs compared to that in all APRs. (Only amino acid residues with at least 50 occurrences are shown.).

To analyze the amino acid sequences of the 173 APR structures, only one representative of the identical amino acid sequences was kept. The remaining 146 amyloid entries of unique sequences consist of 989 amino acids. These oligopeptides were primarily derived from about 40 different proteins, with a few sequences coming from ab initio design. Although the size of this database is not large enough to allow for a proper residue frequency analysis, there are some striking differences compared to globular proteins (Figure 2a): Gly, Asn, Phe, and Tyr are more abundant than in globular proteins; furthermore, amino acids with charged side chains (Lys, Arg, Glu, Asp), with large aromatic side chain (Trp) or with a backbone kink (Pro) are much less abundant. Previous studies on the amino acid composition around or within amyloidogenic segments of proteins revealed categories of aggregation-prone (most hydrophobic residues), aggregation-neutral (mostly polar side chains), and gatekeeper residues (proline and charged side chains) (Figure 2b).^37−39^ However, in the present database, where one would expect enrichment of the aggregation-prone residues, there are some surprising exceptions (Figure 2b), which are discussed below.

1. All aggregation-prone residues are expected to be more frequent in APRs, however, Trp, Cys, Met, and Leu residues, previously identified as aggregation-prone residues,^39^ are underrepresented among these APR structures, which could be explained by the redox sensitivity or by the lower solubility of these residues (Figure 2b, left).
2. Some residues, considered to be aggregation-neutral (Asn, Gly, Ser, Gln) are however overrepresented in these APRs. Both Asn and Gln side chains can form H-bonded ladders that stabilize the β-sheet structures that explain their frequent occurrence in APR structures (Figure 2b) The hydroxyl groups of Ser are also frequently involved in H-bonds, either with solvent molecules or with the free ends of the APRs. The increased presence of the side-chainless Gly could be explained by its flexibility, as it can more easily bend and accommodate the charged N- and C-termini that form H-bond networks in amyloid crystals. The flexibility of Gly may be also advantageous when the backbone adopts kinks in LARKs.
3. As expected, Pro, Asp, Glu, Lys, and Arg, known as amyloid gatekeeper residues, are rarely found in APR crystal structures because they interfere with β-sheet formation (Pro) or with the association of a tightly packed zipper (charged end groups).

Comparing the residue propensities of the parallel and antiparallel APR structures (Figure 2c), we found that both Gln and Asn are preferentially located in the parallel β-sheets, whereas Leu and Ala are more abundant in the antiparallel β-sheets. The former observation can be explained by that Gln and Asn side chains within the parallel structures specifically are in an ideal position to form H-bond-connected side-chain ladders,^40,41^ which network provides additional stability to the amyloid architecture, beyond the interbackbone H-bonds. In addition, ladder-like hydrophobic contacts between alternating side chains, such as Ala and Leu, are often found in antiparallel β-sheets. Similarly, ladders formed by Val-Val or Val-Phe pairs were also found (Figure S1, Table S4). The two different distributions of amino acids also indicate a difference in the hydrophobicities of parallel and antiparallel β-sheet structures. We found that the average hydrophobicity (calculated as the GRAVY index^42^) is lower for parallel than for antiparallel APRs, meaning that parallel β-sheets are less hydrophobic (Figure S2a).

### 2.2 Secondary Structure Analysis Revealed the High Abundance of Parallel β-Sheets

**Figure 3:**
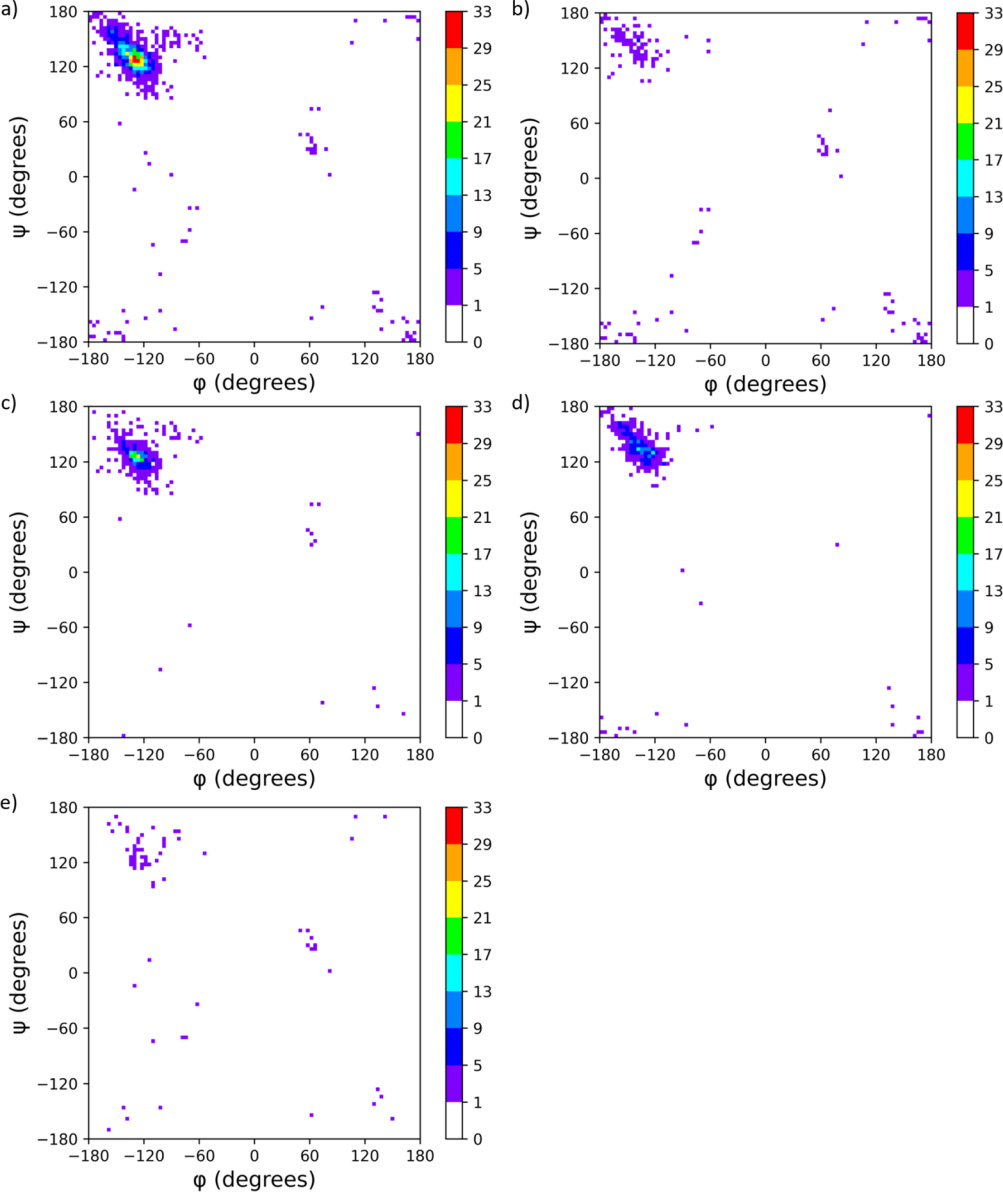
Ramachandran plots reporting on the (φ and ψ) backbone torsion angle pairs of the residues within APR structures. A total of 1272 (φ, ψ) pairs were found in 173 APR structures. (A 4° × 4° bin size data representation.) (a) All residues in APR structures, (b) Glycines only, (c) Parallel β-sheet structures (classes 1, 2, 3, and 4), (d) Antiparallel β-sheet structures (classes 5, 6, 7, and 8), and (e) that of the 13 LARKS structures.

In contrast to globular proteins, the hydrophobic core structures of amyloids are formed almost exclusively by the assembly of β-sheets, consistent with the β-sheet being the dominant structural element of amyloids. Indeed approximately 95% of all residues fall within the allowed β-sheet region of the Ramachandran surface. (Figure 3) Most of the remaining 5% belongs to LARKS. (Figure 3a,e). Moreover, some of the non-β-sheet conformers of APRs are located at their N- or C-termini, are involved in the proper orientation of the adjacent polypeptide termini, and are typically (∼80%) Gly (Figure 3b). However, LARKS structures show that kinked regions are not exclusively formed by glycines (compare Figure 3b,e). On the other hand, segments of extended conformation, with large absolute values of both φ and ψ angles (150°–180°, shown at the corners of the Ramachandran plot (Figure 3a,b) are also rich in Gly.^44^

The main force behind the amyloid structure formation is the molecular packing of the quasi-infinite β-sheets, the conformationally most stable secondary structure element.^45^ The crystal structures of amyloid oligopeptides also consist of these “infinite” β-sheets, which are tightly packed in the other two dimensions. Theoretical studies have shown that antiparallel β-sheets are energetically more stable than parallel ones.^45^ For the most common peptide length in the APR database, hexapeptides, even the number of the backbone H-bonds is higher for the antiparallel β-strands: 10 and 12 H-bonds per polypeptide for an ideal parallel and antiparallel hexapeptide β-sheet, respectively (Figure S3a,b). Although this might predict a higher number of antiparallel than parallel APR structures, unexpectedly parallel β-sheets are more abundant, as we found 104 parallel and 69 antiparallel APR structures (Figure 3c,d, Table S1). Moreover, in full-length amyloids of polypeptides and proteins, this ratio is even more unbalanced in favor of parallel β-sheets, as only two known examples are for antiparallel amyloid organizations.

The above analysis of the ratio of parallel to antiparallel β-sheets within amyloids suggests that the stabilizing factors facilitating amyloid formation are complex. The backbone–backbone H-bonding contacts are only one factor, along with favorable side-chain/side-chain interactions, both within the dry zipper and at the hydrophilic interfaces. These observations have stimulated our interest in quantitatively describing the tertiary structure contacts within different amyloid classes.

### 2.3 Distribution of Topological Classes among APR Structures

**Figure 4:**
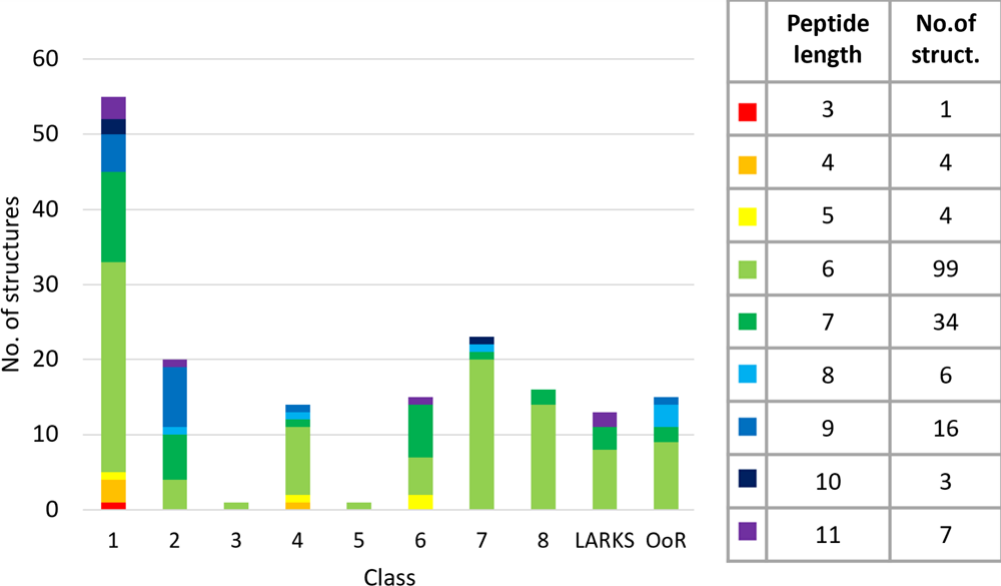
Distribution of the APR structures between topology classes.^27,28,30^ Peptide length is color-coded (with the number of amyloid structures of each length shown). Hexapeptides, being the largest group, are the only group with representatives in all classes. All oligopeptides, except tripeptides, have parallel and antiparallel structures.

Tertiary structure analysis is a comparative study of the amyloid structures belonging to different topological classes, focusing on the orientation of parallel and antiparallel β-sheets, forming different zippers by selecting different sets of secondary interactions. Our analysis revealed (Figure 4, Table S1) that class 1 is the most populated, the one containing parallel antifacial β-sheets, forming two different zipper regions. Classes 2 and 4 of the parallel β-sheets contain less than half as many members. Among antiparallel β-sheets classes 6, 7, and 8 are about equally populated, with class 7 being slightly more populated than the other two. Class 3 (parallel β-sheets) and class 5 (antiparallel β-sheets) each have currently a single representative known, while classes 9 and 10 have none. The two auxiliary classes, OoR and LARKS, are moderately populated. All of the OoR structures are antiparallel, and all but one of the LARKS structures form parallel β-sheets. Although both the OoR and LARKS can be assigned to one of the 8 topological classes (referred to as a pseudoclass in Table S1) based on their β-strand interfaces and orientations, they are treated as separate entities in the analysis below, due to their intrinsic differences.

Classes 9 and 10 (equifacial parallel β-sheets) remain hypothetical, as no representative structure has yet been determined. In both classes 9 and 10, each aligned β-strand has an overhanging residue that is not part of the backbone H-bond network, and therefore, they form much fewer H-bonds than they could in other classes. However, there are examples of overhanging residues depending on the length of the peptide chain. For example, polypeptides of classes 5 and 6 containing an even number of residues (e.g., PDB ID: 6C3T, Figure S3c), or of classes 7 and 8 containing an odd number of residues (e.g., PDB ID: 4W5L). The relationship between stability and the topology-dependent size of the backbone H-bond network is also reflected by the population of different classes: among hexapeptide structures, classes 7 and 8 are more common than classes 5 and 6, while among heptapeptide structures, classes 5 and 6 are the more populated ones. Note, however, that the overhanging residue can be stabilized by forming an OoR β-sheet, where an additional H-bond provides additional stability (Figure S3d). There are several examples of antifacial hexapeptide β-sheets (pseudoclasses 5 and 6 topologies) in the OoR. The out-of-register arrangement of β-strands could in principle stabilize both class 9- and 10-like topologies. Therefore, we suggest that the OoR structure is more likely to form than the class 9 or 10 arrangement of the β-strands.

As discussed above, the stability of the β-sheets alone cannot explain the distribution of the different amyloid classes and the high abundance of parallel β-sheet structures, since the side-chain/side-chain interactions within the dry zippers also play an important stabilizing role. The GRAVY index was used to qualitatively characterize the hydrophobicity of the different interfaces formed by the two faces of the β-sheets. We have calculated the difference of the GRAVY indices of residues 1,3,5 and 2,4,6 of the hexapeptide structures (see Section 4 and Figure S2b). These two sets of residues are collected in separate interfaces in structures of classes 1, 3, and 5. GRAVY calculated for these three classes shows a bimodal distribution (Figure S2b) indicating that there are structures with interfaces of similar polarity (small differences in GRAVY indices), while in other structures there are separate hydrophobic and hydrophilic interfaces within the same structure (large differences in GRAVY indices). In classes 2, 4, and 6 these two sets of residues together form each interface–which does not allow for the separation of polar and apolar surfaces, in accordance with the unimodal distribution of the difference-GRAVY values. In classes 7 and 8, where each face of the β-sheet contains all the residues, most structures show a small difference-GRAVY values. Here, many peptides consist exclusively of hydrophobic amino acids.

### 2.4 Tertiary Structure: Quantifying the Inter-β-Sheet Interactions

A different approach to describing molecular recognition is to characterize the steric fit and size of the complementary surfaces that form zippers. The interactions between the β-sheet elements in APR structures are similar to those of the full-length amyloids formed during the process of water exclusion and fibril formation. This can also be interpreted as the sandwich-like tertiary structure of amyloid oligopeptides, formed by stacked β-sheets. Two descriptors are typically used to characterize the inter-β-sheet contacts manifested by side-chain/side-chain interdigitation within the interface: shape complementarity (Sc) and buried area (Ab). The values of both descriptors strongly depend on the number of β-sheets considered in such a calculation, since (i) the outermost chains have different van der Waals surfaces, lacking a connected β-strand on one side, (ii) Ab increases with the size of the system considered, and (iii) each structural element may be considered a different number of times in the case of structures with two β-strands per repeating unit. However, the number of β-strands for which these values have been calculated is scarce in the literature. We propose here an automated and unified method to calculate the descriptor values for a thorough analysis of the database. (i) The number of the β-strands in the interface-forming β-sheet-pairs should be three times that in the repeating unit (three β-strands for parallel and six β-strands for antiparallel β-sheets, see Section 4 and Figures S4 and S5). (ii) Instead of the total buried surface area, Ab is calculated for the middle β-strand(s) of the segment of β-sheets used for the calculation, and then this value is related to one peptide chain (Figure S6). (iii) Contact regions of only the zipper interfaces are considered: we introduced a lower limit of the solvent-excluded area of the peptide chains involved, and criteria for the involvement of side chains (see Section 4).

**Figure 5:**
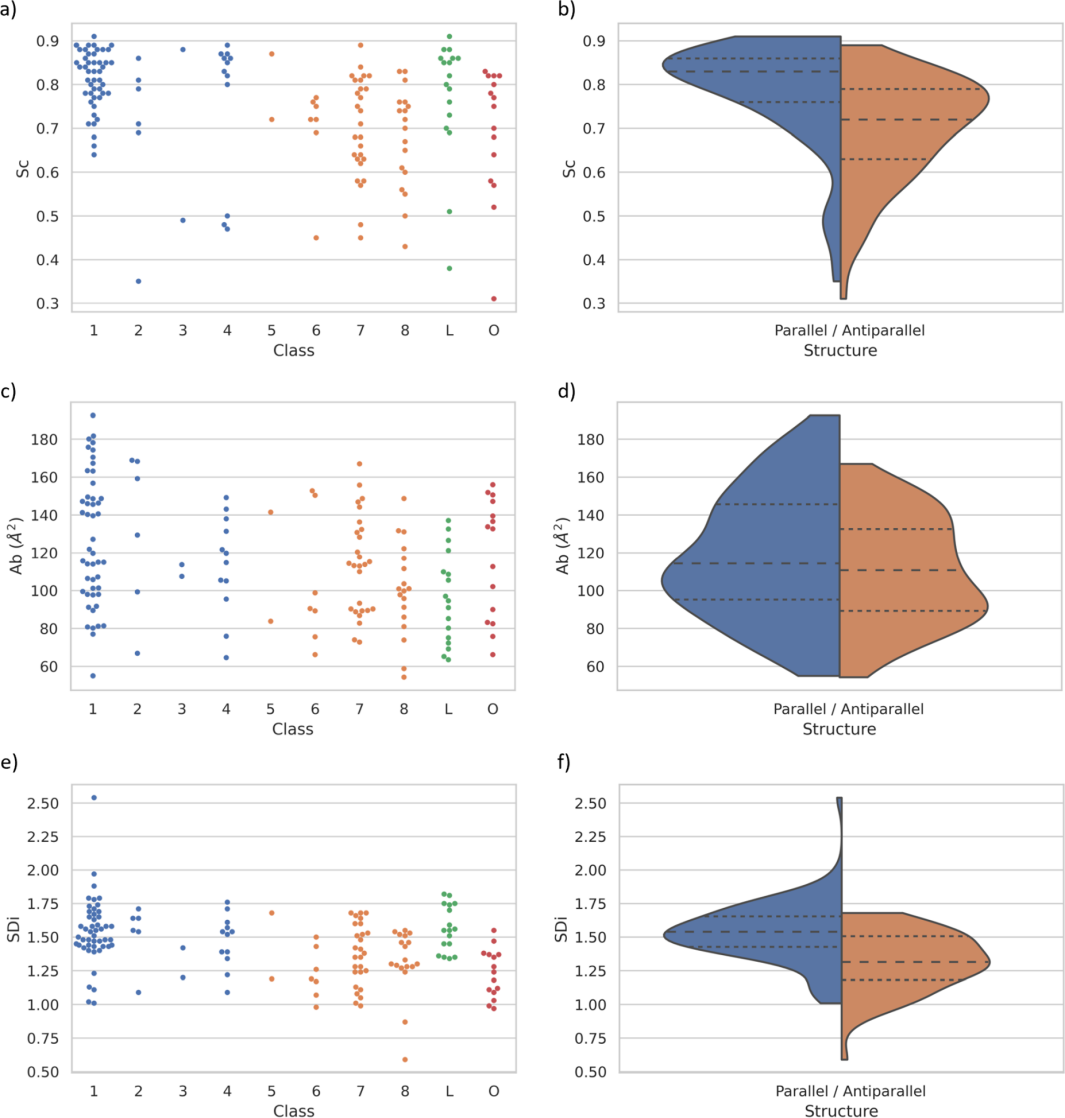
Characteristics of interfaces between β-sheets as reported by the 3 descriptors, Sc, Ab, and SDi used for amyloidogenic oligopeptide structures (shown for hexapeptides). (a, c, e) Distribution of descriptor values for each topological class as swarm plots. (b, d, f) Comparison of the distributions for parallel (classes 1–4, LARKS) and antiparallel (classes 5–8, OoR) structures (violin plots). Horizontal dashed lines show the median and interquartile range.

Among the 173 amyloid structures studied, there are 267 distinct zipper interfaces (descriptor values are compiled in Table S3). Interestingly, about a quarter of them are partially wet interfaces (i.e., solvent molecules are trapped between the β-sheets, breaking the dry interface). However, wet interfaces^24^ where the solvent covers the entire side of a β-sheet do not meet the above criterion and are not discussed here. The analysis of APR zipper interfaces is shown in Figure 5 for the most populated group, hexapeptides, for better comparability. (Analysis for different oligopeptide lengths gave similar results, Figure S8.) Sc and Ab allow the comparison of complementary properties of the interfaces, indicated by their weak correlation (*r* = 0.23 for all structures, Figure S8b). The descriptor values of the different interfaces are highly variable, but some notable trends were found as follows.

Analysis of the Sc values revealed that (i) the distribution of Sc is broad with overlapping unimodal distributions of parallel and antiparallel structures (0.75 ± 0.13; Figure 5a,b). (ii) Notably Sc of parallel APR structures tends to be higher: it is greater than 0.76 for 75% of the interfaces of parallel structures, also higher than the average values of high-affinity protein–protein complexes. Reference values considered are the protease-inhibitor complexes (0.73 ± 0.03) and antibody/antigen complexes (0.66 ± 0.02).^46^ (iii) Partially wet interfaces tend to have significantly low Sc values (below 0.6). (iv) Shape complementarity is independent of the oligopeptide chain length (Figure S8b).

The analysis of the Ab values led to less general conclusions, showing that Ab rather depends on the type of side chains forming the interface (even if only values for hexapeptides are compared, Figure 5c,d): (i) The average of Ab is 115 ± 31 Å^2^ (with the parallel structures having slightly higher values: 119 and 110 Å^2^, respectively). (ii) They show an overlapping bimodal distribution, more pronounced for antiparallel structures, corresponding to interfaces with different properties. The smaller interfaces (60–120 Å^2^) are formed by fewer side chains or are less interdigitated (typical amino acids: Val, Ala, Ser, Gly), or have solvent molecule(s) included in the interface (partially wet interfaces). Larger solvent-excluded surface areas (120–200 Å^2^) correspond to highly interdigitated surfaces with longer side chains that connect in a cog-like fashion (e.g., Gln, Asn, Leu, Lys). (iii) The comparison for different topological classes showed similarly wide distributions, with LARKS structures tending to have the smallest interfaces. The latter could be explained by considering the kinked β-sheet-like structures of LARKS oligopeptides as they can bury some of their side chains excluding them from the interface. (iv) The analysis of APR structures of different lengths shows, as expected, that Ab correlates with the length of the oligopeptides. Interestingly, however, the tetrapeptides do not follow this trend with average Ab values closer to those of the hexapeptides (Figure S8c).

### 2.5 Tertiary Structure: Landscape of Amyloid Interfaces Characterized by the New Descriptor SDi

**Figure 6:**
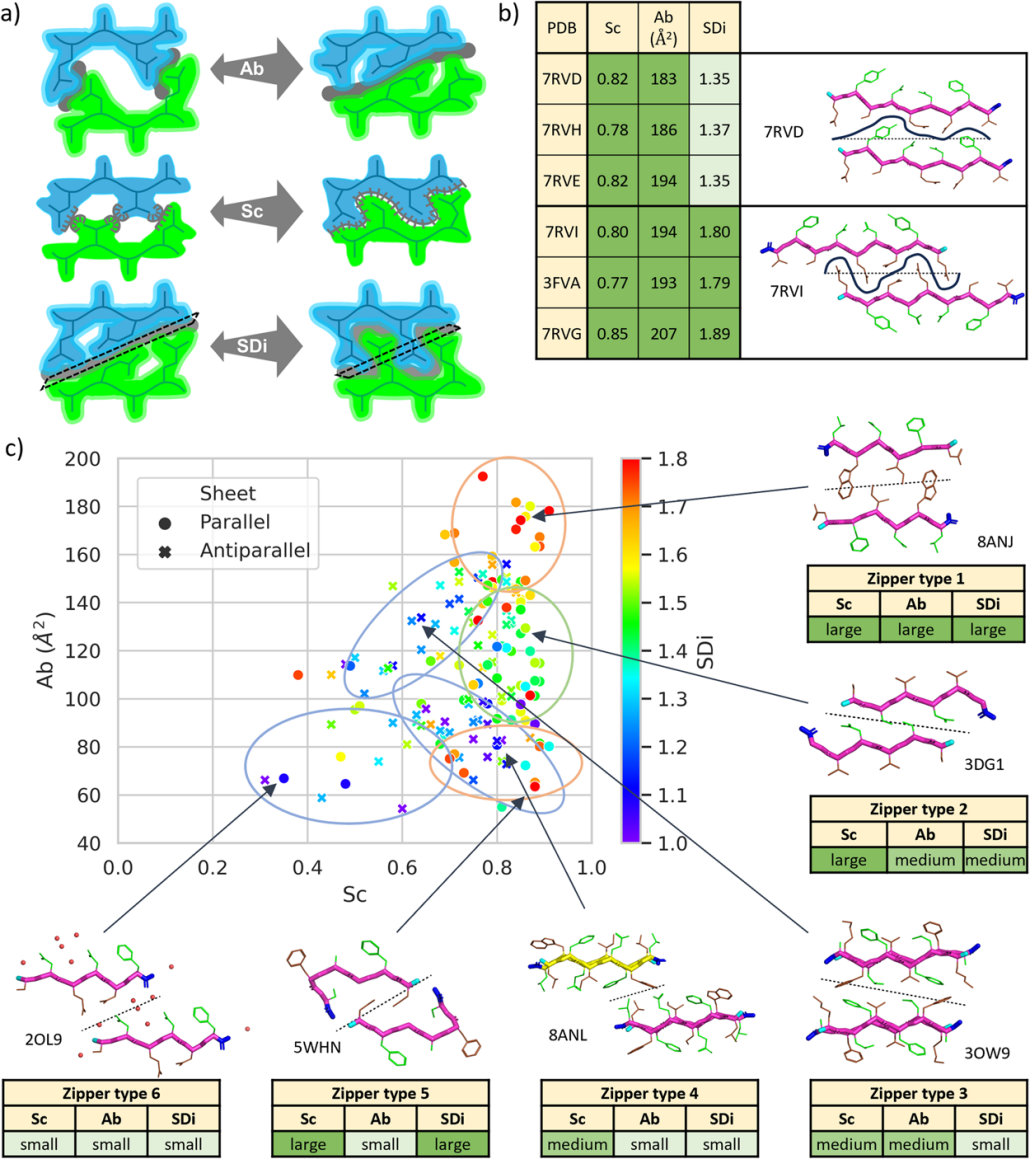
SD_i_ represents the smoothness or cog-like characteristics of the zipper interfaces formed between β-sheet pairs. (a) The schematic representation of the definition of Sc, Ab and SDi. (b) An example of the complementarity of Sc, Ab and SDi descriptors in the characterization of amyloids. Only SDi differentiates between the interface families with different H-bond networks, as shown by the example of the mammalian prion β2α2 loop APRs.^47^ (c) Co-distribution of amyloid interface descriptors in hexapeptide structures (the 3D space is shown as an Ab vs Sc plot, with SDi values color-coded). The 6 clusters of different zipper interfaces with examples for each one (PDB IDs are labeled).

Since amyloid formation involves the step of water exclusion from and between the matching β-sheet surfaces, self-recognition and 3D complementation of the amyloidogenic oligopeptides is mandatory.^11,25^ While Sc describes the fit between the curved surfaces and Ab measures the area of the interface, neither can distinguish the interdigitation or flatness of the matching interfaces. Here we propose a new descriptor, the Surface Detail Index (SDi), which is the degree of interdigitation. It is defined as the ratio between the buried surface area of an interface and the area of its flat “footprint” (Figures 6a and S7). The “footprint” here is a rectangle: the interface is projected along the axis of the β-sheet (*y*-axis as shown in Figure 1) to get the length of the rectangle, while the height of the rectangle is the distance between the β-strands within the β-sheet. SDi is independent of the length of the polypeptide. Its typical value is around 1.45 for moderately wavy interfaces, which increases to 1.8 for highly detailed and interdigitated interfaces (Table S3).

Analysis of the SDi values in the APR structural database (Figure 5e,f) leads to the following general findings. (i) The average value of SDi is 1.45 ± 0.23, which shows that the zipper interfaces are generally 45% larger than a flat surface of a similar footprint would be. (ii) SDi shows weak correlations with both Sc and Ab values (*r* = 0.30 and 0.31, respectively for all structures), suggesting that SDi reveals and describes a new feature of the zipper interface, complementary of the other two descriptors. (Figure S8b,d,f) (iii) The distribution of SDi values revealed, that the interfaces of antiparallel β-sheets tend to be flatter (average SDi of cca. 1.32) compared to those of parallel topologies (average SDi of cca. 1.53). (iv) No SDi trends were found between the different topological classes, although LARKSs tend to have larger than average SDi values, suggesting that the bending of the backbones in LARKS structures creates a more detailed and more interdigitated surface. (v) By analyzing the tetrapeptide amyloids, we found that their SDi values are higher than calculated for longer polypeptides (Figure S8e), so their higher-than-expected buried surface area can be explained by their more interdigitated interfaces.

To illustrate the ability of SDi to discriminate between different interfaces of homologous polypeptides (a feature of amyloids that could be considered as polymorphs of the zipper interfaces), we calculated the Sc, Ab, and SDi values for the β2α2 loop structures of the mammalian prion protein (Figure 6b). Glynn et al.^47^ identified two different packing modes of the β-sheets induced by sequence variations and forming either class 1 or class 2 zipper interfaces. Note that the two different topologies are stabilized by different H-bond patterns and different side chain conformers of the QXN motif (Figure 6b). The two zippers look different, as the first (PDB: 7RVD) is quite flat, while the second one (PDB: 7RVI) is jigsaw-like. While both the Sc and Ab values of the two zippers are somewhat similar, indicating that both types of packing result in a similarly tight fit of the surfaces, only their SDi values indicate that they belong to two different polymorphs. The first type of packing has a lower SDi value (∼1.36) signaling a less interdigitated interface, compared to the second one (∼1.84).

Using these three complementarity descriptors, Sc, Ab, and SDi, the fine structure of the zipper interfaces within all APR structures can now be properly represented in three dimensions. Clusters of distinctly different interfaces can be identified as distinct regions of such a 3D plot (Figure 6c), showing that they would not be separable using only the 2D plot of the two descriptors: (Sc, Ab). We found 6 clusters representing 6 characteristically different inter-β-sheet or zipper interfaces.

***Zipper type 1*** is an interface where all the 3 descriptors have high values. These typically parallel β-stranded structures are densely packed with highly detailed zipper interface surfaces where larger side chains are present. ***Zipper type 2*** interfaces are typically formed between parallel β-sheets and can be characterized by medium Ab and SDi values and large Sc values. These surfaces are typically composed of smaller side chains that are unequally interdigitated. ***Zipper type 3:*** These interfaces have medium Ab and Sc values and smaller SDi values. They are typically formed by large and noninterdigitated side chains. The β-sheets are most often antiparallel. ***Zipper type 4*** contains interfaces with small Ab and SDi and medium Sc values. In this case, shifted β-sheets are often formed, resulting in two partial interfaces. The cluster of ***Zipper type 5*** is typically observed in LARKS of highly kinked backbone conformers forming interfaces with larger SDi and Sc values but small Ab values. ***Zipper type 6*** interfaces have lower values for all three descriptors, forming partially wet interfaces. In these cases, water or other solvent molecules are incorporated through the interface or the terminal groups of the peptide have a significant contribution to the interface.

Finally, we found that some regions of the 3D plot are poorly populated or empty (Figures 6c and S8, shown for hexapeptides). (i) We have not yet assigned interfaces with large Ab, but small Sc values, which would correspond to loosely packed large interfaces. Such an interface would have gaps smaller than solvent molecules, which is unlikely in a crystal structure. (ii) At a given peptide length, Ab and SDi values show some correlation: larger interfaces of a certain footprint can be achieved only by more interdigitated side chain interactions, so interfaces with large Ab - small SDi value pairs are not present in the database. (iii) The combination of small Sc and large SDi must be unfavorable, as large side chains would interact in poor complementarity positions.

### 2.6 Quaternary Structure as Networks of Interfaces

**Figure 7:**
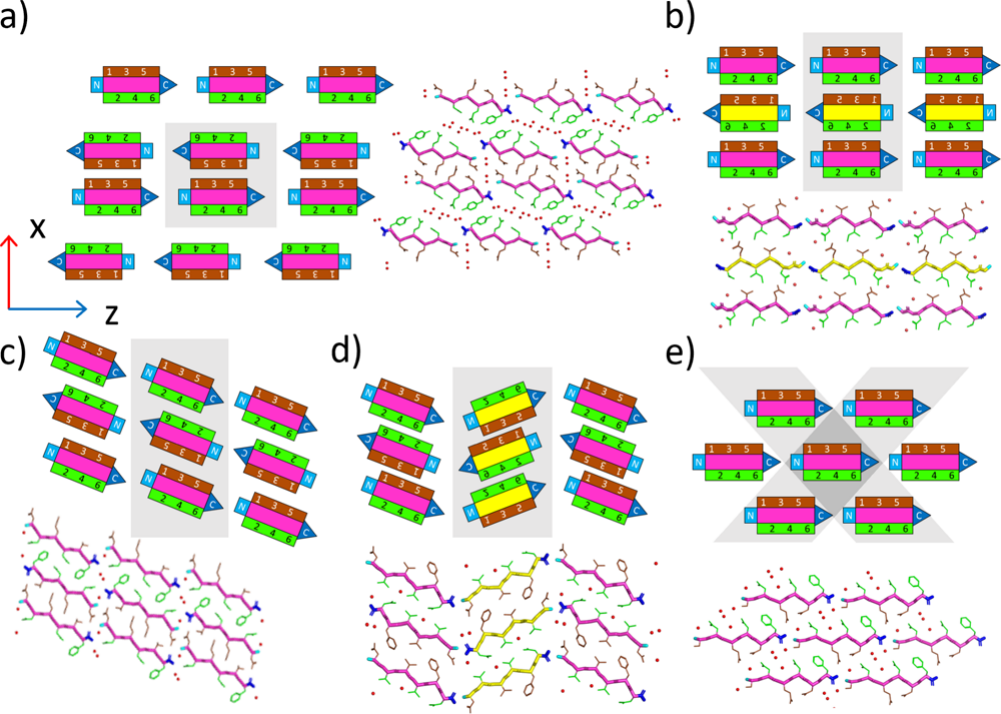
Quaternary level of amyloid structures is the crystal packing of β-sheets. The repeated pattern of β-sheet pairs forming interfaces can be viewed as a network of zipper connections. These connections can form 1D (a), 2D (b–d) or 3D networks (e) (shown in gray). Schematic representations are shown using parallel β-sheet structures as examples. (a) 1D network contains pairs of interacting β-sheets surrounded by water molecules. (b–d) 2D network containing a series of β-sheets (layer) forming interfaces with each other–these networks can be further characterized based on shift along *x*-axis, or tilt around *y*-axis: (b) straight (c) tilted (d) fishbone. (e) 3D network with brick wall pattern (PDB IDs: (a) 1YJO (b) 3SGS (c) 3FVA (d) 6RHA (e) 2OL9).

The quaternary structure of the amyloid describes the manner in which its tertiary structure elements interact, either in the crystal or in the fibers. This nanoscale molecular packing is determined by the repeated patterns of interaction between the many β-sheets, held together by zippers (dry or partially wet), and the termini. Considering a typical amyloid arrangement in APR structures (Figure 1), if the β-sheets are parallel to the *y*-axis (Figure 7) then the quaternary interactions connect individual β-sheets in the (*x*,*z*)-plane, the latter include interactions of the zipper interfaces (general direction *x*) as well as interactions of the charged termini. The complex network of amyloid quaternary structure can be subdivided into a 1D-, 2D-, or 3D- network of interfaces, formed between β-sheets (Figure 7). In the case of a 1D network, each zipper-joined β-sheet pair (i.e., the tertiary structure element) forms a single β-sandwich (Figure 7a), surrounded by extended solvent regions, forming distinct dry and wet interfaces. This type of network could be weak, with “core elements” that easily slide over each other, and is therefore found in only about 10% (15 structures) of all the crystalline cases analyzed here. On the contrary, in more than 70% (125 structures) of the cases analyzed, the quaternary structure is held together by 2D networks. In general, both faces of β-sheets each form a dry zipper interface, and thus these assembled core elements form an infinite and aligned series of β-sandwiches (catemer synthons, Figure 7b), aligned along the *x*-axis. These layered superstructures are lined up together as 2D networks separated by the N- and C- termini of the peptide chain, which often include solvent molecules. When the interfaces are laterally displaced within the same layers, the packing pattern can be either straight or tilted (Figure 7b,c). The straight pattern is more common in classes 4 and 8, where the internal symmetry favors only small displacements. If there is a crystallographic screw axis parallel to the *z*-axis, then the tilted pattern can form an alternating fishbone pattern (Figure 7d).

Where the lateral displacement of the β-sheets within the layers is large (nearly half the length of the oligopeptide chain length), each β-sheet forms more than two interfaces in the crystal lattice; thus, a 3D network of interfaces is created. We found 33 such amyloid structures. In these structures, the polypeptide chain ends are sandwiched between the surface of other β-sheets, forming buried ion pairs (brick pattern, Figure 7e). In some cases, the interfaces can belong to different topological classes (see the Supporting Information for details). Due to the staggered arrangement, the interfaces of the 3D networks have lower than average Ab values, but similar interdigitation as in 2D networks is possible, as shown by the SDi distributions (Figure S10). The value of Sc tends to be lower because in many cases the terminals are also part of the interface, often forming ionic interactions sterically less favorable but stronger.

LARKS structures generally do not follow the above networking patterns because their kink causes the terminals to be oriented in a nonopposite direction. They usually form a tilted 2D network pattern, or a basket weave pattern, which can be considered as either a 2D or a 3D network (Figure S11). Some APR oligopeptides crystallize with three- or six-fold symmetry (Figure S12; Table S2). In these structures, the β-sheets form nanopores. Due to significant solvent channels and the lack of comparable interfaces on both sides of the β-sheets, these structures are beyond the scope of this study.

### 2.7 Analyzing Polymorphic Structures Based on Structural Hierarchy

There are currently 19 APR sequences in the database with known polymorphic structures. Polymorphs are formed depending on solvent composition, temperature, and aging (just to mention some of the major factors). In these polymorphs, often different sets of interactions stabilize the zippers, and the topologies can be also drastically different (e.g., parallel and antiparallel structures). To present the tool-set of ACW in comparing the steric features and topologies of polymorphs we show here the recent example of crystal structures of the amyloidogenic oligopeptide of Tc5b miniprotein (Table 1), which has the largest number of polymorphic structures reported.^48^ The three descriptors also help understand which inter-β-sheet interface can be considered the major or minor site of organization of individual β-sheets to a mesostructure.

**Table 1:** Comparison of Steric Parameters of the Amyloid Polymorph Structures of Oligopeptide Leu-Tyr-Ile-Gln-Trp-Leu (Individual Residues Are Referenced with One-Letter Code and Sequential Numbers)

Crystallographic parameters^a^			Topology and descriptors of the zippers^b^						
PDB code	Space group	Unit cell volume; volume per peptide chain (Å^3^)	Class	Class of interfaces	Residues forming the two sides of interfaces	Sc^c^	Ba (Å^2^)^c^	SDi	Quaternary structure
8ANG	*P*2_1_2_1_2_1_	4712; 1178	1 parallel	14	L_1_I_3_W_5_: L_1_I_3_W_5_	0.64	98.0	1.51	3D
					Y_2_Q_4_L_6_: W_5_	0.48	64.6	1.11	
8ANH	*P*2_1_	5091; 1273	OoR antiparallel	8	L_1_I_3_W_5_,Y_2_Q_4_L_6_: L_1_I_3_W_5_,Y_2_Q_4_L_6_	0.70	139.5	1.18	2D
8ANI	*P*2_1_	5160; 1290	OoR antiparallel	8	L_1_I_3_W_5_,Y_2_Q_4_L_6_: L_1_I_3_W_5_,Y_2_Q_4_L_6_	0.64	133.8	1.12	2D
8ANM	*P*2_1_2_1_2_1_	4873; 1218	8 antiparallel	8	L_1_I_3_,Q_4_L_6_: Y_2_,I_3_W_5_	0.70	86.0	1.31	3D
8QWW	P2_1_	4396; 1099	4 parallel	4	Y_2_Q_4_L_6_: I_3_W_5_	0.82	138	1.78	2D

a Data extracted from the PDB.^13^

b Data generated by the ACW tool.

c Calculated using programs AREAIMOL and SC from the CCP4 package.^49^

The “dryest” of all five known structural variants is PDB structure 8QWW, indicated by the smallest volume of the unit cell and the smallest number of solvent molecules present (2 water molecules per unit cell). While the buried area of the interfaces is not the largest one, Sc and SDi values are the highest in this comparison, meaning solvent exclusion is realized by the best steric fit and interlocking of surfaces.

Structure 8ANG belonging to topological class 1 is a good example of the formation of two interfaces significantly different in all characteristics: the larger interface makes up a hydrophobic core-like region containing only hydrophobic side chains. The smaller interface contains side chains Tyr and Gln forming an H-bonded network along the β-sheet. This minor interface shows a different topology, class 4, and its composition is similar to that of 8QWW, however, there are ethanol molecules included in the interface here, resulting in small values of all three descriptor values.

Descriptor values of the interfaces are also capable of showing small differences in similar structures. For example, the two isostructural structures (8ANH and 8ANI) only differ in the solvent type filling the cavity lining the edge of the zippers. The smaller volumes of these solvent molecules (acetonitrile instead of ethanol) allow tighter packing and smaller unit cell volume in the case of 8ANH. The higher descriptor values of the interfaces of 8ANH indicate that this also results in better steric fit within the zipper regions.

## 3 Conclusions

Amyloid-prone regions, APRs, are thought to be the key elements in the transformation of folded or unstructured proteins into large β-structures containing amyloid fibrils and fibers. In the present study, we have introduced a new tool (ACW) for the comparative analysis of the composition and topological and steric features of APR structures. The new descriptor of the zipper interfaces, the surface detail index, (SDi), characterizes the degree of entanglement of interacting side chains, which is thought to play an important role in molecular recognition of β-sheet surfaces and in the extension of the structure in directions perpendicular to the β-sheet axis. The redefinition of the shape complementarity (Sc) and solvent-excluded surface area (Ab) and the introduction of surface detail index (SDi) descriptors provide a tool for quantifying the different properties of the zipper interfaces, which are considered key structural elements for the exclusion of water from the complementary β-sheets during amyloid formation.

Although theoretical studies have shown that the backbone H-bond network of the antiparallel β-sheet is more stable, parallel β-sheet structures seem to be more abundant (61%) in the current amyloid data set analyzed. We found that both the sequence- and side-chain-governed interactions of the zipper interfaces are key factors in stabilizing the amyloid mesostructures in water. The zipper interfaces of the parallel amyloid structures with similar average buried surface areas to antiparallel β-sheet comprising amyloids have higher shape complementarity and surface detail index, indicating that the interfaces of parallel amyloids have a “better fit” and “deeper entanglement”, which could be a clue for their higher stability.

Our results show that interestingly glycine is the most abundant amino acid in amyloid of APRs, although it is considered as an aggregation-neutral residue. A possible explanation for its high abundance is that as a side-chainless and highly flexible reside^11,50^ it can facilitate the proper orientation of the charged N- and C-termini when aligning the β-sheets of amyloids. As expected, the aggregation-prone hydrophobic residues are abundant in antiparallel APR structures. However, in parallel APR structures, the aggregation-neutral Asn is more abundant than the hydrophobic residues. As previously proposed,^51^ hydrophobic (aggregation-prone) residues promote the formation of amorphous cross-β structures, whereas highly structured amyloid fibrils require polar side chains too (e.g.–CONH_2_ groups) such as those of the aggregation-neutral Asn and Gln. The Asn or Gln side chains form an intra-β-sheet ladder, specific to the in-register arranged parallel β-sheet, thus further stabilizing these structures. A further factor making the formation of antiparallel organization of polypeptides in matured amyloid forms unlikely is that the polar and apolar clusters of residues should be in somewhat palindromic order to form favorable interactions in antiparallel β-sheets. In the initial steps, however, the formation of short antiparallel β-sheets is possible and can be stabilized by hydrophobic ladder-like interactions seen in APR structures.

The dynamic process of amyloid formation and disassembly, as well as aspects of related conformational stability and unfolding, have been studied by molecular dynamics calculations for various systems (currently reviewed in ref (52)). The three descriptors could also be useful in analyzing snapshots of molecular dynamics simulations with all-atom models, complementing the interaction energy calculations with the structural-topological characterization of the zipper interfaces. Such analysis could help us to understand the evolutionary processes of the mesostructures by characterizing partially disassembled and metastable associates or by comparing segments of longer fibril structures. Amyloid fibrils and fibers both show considerable polymorphism. The formation and maturation of their full-length amyloid structures may involve several transient structures, perhaps of different topologies.^50^ The present study provides a tool for analyzing the interfaces of APR crystal structures, and model systems for exploring basic elements of self-complementarity of zipper regions and understanding their self-assembly through different topologies. These results not only help understand the fine details of molecular recognition during self-assembly and distinguish between major/minor sites of organization; but could also provide clues for the design of new materials of special properties based on amyloid-like assemblies. Our work can also be viewed as a proof-of-concept study; the new tools we provide will allow a more straightforward analysis of amyloid aggregates and their rational design.

## 4 Methods

### 4.1 Extraction of Amyloid Core Structures from the PDB

Structures containing an infinite β-sheet motif (determined either by X-ray or electron diffraction methods) were extracted from the PDB.^13^ 217 structures were found, consisting of oligopeptides of 3–11 amino acid residues (Tables S1 and S2). 173 unique structures were analyzed (Table S1), 43 of the structures were excluded from further analysis (Table S2) for the following reasons: (i) racemic peptides (8 structures); (ii) irregular topologies of the interfaces or no interface (8 structures; 5 of them are structures of trigonal or hexagonal organization, discussed in Figure S12); (iii) a structure containing a post-translational modification, or (iv) they are isostructural duplicates of other structures (25 structures). The topological classification was done according to the literature, 1–8 classes,^25^ 2 auxiliary classes (Out of register^28,46^ and LARKS^30^), and unique examples. No examples of classes 9 and 10 have been found.^27^

### 4.2 Structure Analysis Software

The structures were analyzed using Pymol^35^ (visualization and symmetry expansion), SC^32,53−55^ (Sc, shape complementarity index calculation) and AREAIMOL^33,56^ (SASA, solvent excluded surface area calculation) from the CCP4 package,^49^ and BioPython^57^ (backbone dihedral angle calculation), and assignment to Ramachandran regions was performed according to Molprobity.^58^ To automatically generate input files and find interfaces, we developed a plugin for PyMol called the Amyloid Coordinate Wizard (ACW). It contains the database of APR structures and their interface data analyzed in this study, as well as a tool for the visualization of crystal packing, identification of interfaces, calculation of Ab, Sc, and SDi descriptor values using CCP4 programs,^49^ and analysis of topologies of individual interfaces also for new entries. Plots were generated using Matplotlib^59^ and Seaborn.^60^

### 4.3 Method for Selecting Interfaces of Side-Chain Interactions

ACW plugin uses the PyMol symmetry expansion tool to expand the structure, and then peptide chains are assigned to β-sheets based on inter-backbone H-bonds. For the collection of all types of interfaces, the β-sheets are checked for contacts with all other β-sheets. We define the interfaces of β-sheet pairs for further analysis that meet all of the following criteria: (i) size limit: the buried surface area must be at least 50 Å^2^; interdigitation requires involvement of side chains: (ii) each β-strand has at least one side chain that is part of the interaction and (iii) at least an average of 1.5 side chains per a peptide chain should be involved in the interface (making contacts with the meeting β-sheets); and (iv) size limit for assigning a side chain to the interface: a side chain is only considered to be involved if it has at least 10 Å^2^ Ab, in the case of glycine the CA atom must have at least 5 Å^2^ Ab. In this study, only contacting β-sheet pairs meeting the above four criteria were used to analyze the descriptor values Ab, Sc, and SDi (although it is possible to calculate them for other interfaces too using ACW). (Note that hydrogen atoms and atoms of alternative conformations are deleted prior to calculations as they would interfere with the calculation of the descriptors.) A structure may contain the same interface several times either due to crystallographic or noncrystallographic symmetry elements. Descriptor values for interfaces with the same interacting side chains and the same topological classes are averaged. Using these criteria, we found 267 distinct zipper interfaces in the 173 structures.

### 4.4 Uniform Definition of the Structure Size and Analysis of Amyloid Interfaces

1. Shape complementarity (Sc) was calculated with the SC program, using default settings. It uses the buried molecular surfaces with a cutoff distance of 1 Å. Normal vectors are calculated using a grid of 0.1 Å resolution for the molecular surfaces of both β-sheets, and the Sc value is the median of the scalar products of the normal vector pairs. Since this calculation method does not allow the calculation of the Sc of a part of the structure (i.e., the middle repeating units), there is a bias in the descriptor values calculated for the outermost peptide chain of a finite β-sheet. To minimize this, we used 3 repeating units of the β-sheets (i.e., 3 chains for parallel and 2 chain pairs for antiparallel structures) to calculate the Sc value (Figure S4). Further increasing the system size causes only very small changes in the standard deviation of Sc values (Figure S5a). We also identified eight out of all interfaces for which Sc calculated for the proposed structure size deviates from the estimated Sc value for a large structure by more than 0.03 (Figure S5b).
2. The buried surface area (Ab) is the averaged lost area of solvent accessible surface area (SASA) of one peptide chain buried in the interface. The previous distortion can be completely circumvented by calculating the SASA of only the middle repeating units of the two β-sheets (the same 3 repeating units each as above) forming the interface: SASA is calculated for the two β-sheets separately (Figure S6 SASA_A_, SASA_B_), and also SASA of the β-sheet (SASA_U_). Their difference is the buried area of the middle repeating unit, which should be divided by the number of chains within the two repeating units (*N* = 2 and 4 for parallel and antiparallel β-sheets, respectively) to obtain the average value for a β-strand (eq 1)
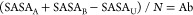
1
3. A new descriptor was created to distinguish differently interdigitated structures, called the Surface Detail Index (SDi). It is calculated as the ratio of the buried surface area of the interface and the area of a rectangle, which approximates a flat surface of a similar footprint (Figure S7). The width of the rectangle is the width of the repeating unit (*y*-axis direction, calculated from the distance between the centroids of the β-strands). To determine the length, first, a projection of the structure along the β-sheet axis (*y*-axis) is created, then the positions of the probe circles (water molecule, 1.4 Å) are determined where they touch the projections of both β-sheet surfaces (similar to the calculation of the solvent accessible surface, but in 2D). The endpoints of the “length” are determined as the innermost facing point of the probe circle between the tangent points. (Figure S7) (Note,that because of the irregular shape of the interface's edges, the length of the rectangle is an upper approximation of the actual size of the “flattened” interface.)

## Data Availability

ACW plugin for PyMol^35^ (including database of the APR oligopeptide structures and resulting data of their analysis presented here) is made publicly available on GitHub: https://github.com/MateSulyokEiler/ACW.

## Supporting Information Available

Secondary interactions and residue characteristics within β-sheets; supplementary methods; additional analysis; and supplementary results (PDF)
PDB structures of APR oligopeptides analyzed (XLSX)
PDB structures of APR oligopeptides excluded from the present analysis (XLSX)
Interfaces and descriptor values of APR oligopeptide structures (XLSX)

## List of abbreviations

**Ab**: buried area of the molecular surface
**ACW**: Amyloid Coordinate Wizard is a PyMol plugin to analyze amyloid structures, presented in this article
**APR**: aggregation prone region
**LARKS**: low-complexity aromatic-rich kinked segments
**OoR**: out-of-register β-sheet
**PDB**: Protein Data Bank
**Sc**: shape complementarity
**SDi**: surface detail index
**SASA**: solvent accessible surface area
