# Supplementary material for: Unravelling the Complexity of Amyloid Peptide Core Interfaces
Source: J Chem Inf Model. 2024 Oct 30;64(22):8628–40. doi: 10.1021/acs.jcim.4c01479 (PMC11600497; doi:10.1021/acs.jcim.4c01479)
Supplement: Supplementary file 1 — ci4c01479_si_001.pdf [file ci4c01479_si_001.pdf]

Supporting Information

Unravelling the Complexity of Amyloid Peptide Core Interfaces

*Máté Sulyok-Eiler[2,3], Veronika Harmat[2,4] and András Perczel[1,2,4]**

1 Medicinal Chemistry Research Group, HUN-REN Research Centre for Natural Sciences, Magyar Tudósok Körútja 2, H-1117 Budapest, Hungary.

2 Laboratory of Structural Chemistry and Biology, Institute of Chemistry, ELTE Eötvös Loránd University; Pázmány P. stny. 1/A, H-1117 Budapest, Hungary

3 Hevesy György PhD School of Chemistry, Institute of Chemistry, Eötvös Loránd University; Pázmány P. stny. 1/A, H-1117 Budapest, Hungary

4 HUN-REN-ELTE Protein Modeling Research Group, Hungarian Research Network; Pázmány P. stny. 1/A, H-1117 Budapest, Hungary

Index

Table S1 – PDB structures of APR oligopeptides analyzed in this study. (It can be downloaded as a separate file.)

Table S2 – PDB structures of APR oligopeptides excluded from the present analysis. (It can be downloaded as a separate file.)

Table S3 – Interfaces and descriptor values of APR oligopeptide structures. (It can be downloaded as a separate file.)

Secondary interactions and residue characteristics within β-sheets 2

- **Figure S1.** Examples of amino acid ladders 2
- **Table S4.** Interacting amino acid pairs of neighboring β-strands of antiparallel APR structures 2
- **Figure S2**. Hydrophobicity of APR structures shown as distributions of GRAVY index 3
- **Figure S3**. Comparison of in-register and out-of-register antiparallel H-bond patterns 4

Supplementary Methods

- **Figure S4.** Dependence of calculated Sc and Ab values on system-size. 5
- **Figure S5.** Dependence of the bias of Sc (caused by the outermost peptide chain) on system size. 6
- **Figure S6.** Structural regions for calculating surfaces 7
- **Figure S7.** Definition of SDi. 7

Additional analysis of Sc, Ab and SDi distributions

- **Figure S8.** Ab, Sc and SDi plots for different peptide lengths; and their correlation 8
- **Figure S9.** Co-distribution of amyloid interface descriptors in hexapeptide structures, projections. 9

Supplementary results for APR quaternary structure 10

- **Figure S10.** Distribution of Sc, Ab and SDi values for different quaternary structure topologies 11
- **Figure. S11.** Non-typical quaternary level structures (examples). 11
- **Figure S12.** Trigonal or hexagonal structures of APR oligopeptides. 12

References 12

Secondary interactions and residue characteristics within β-sheets

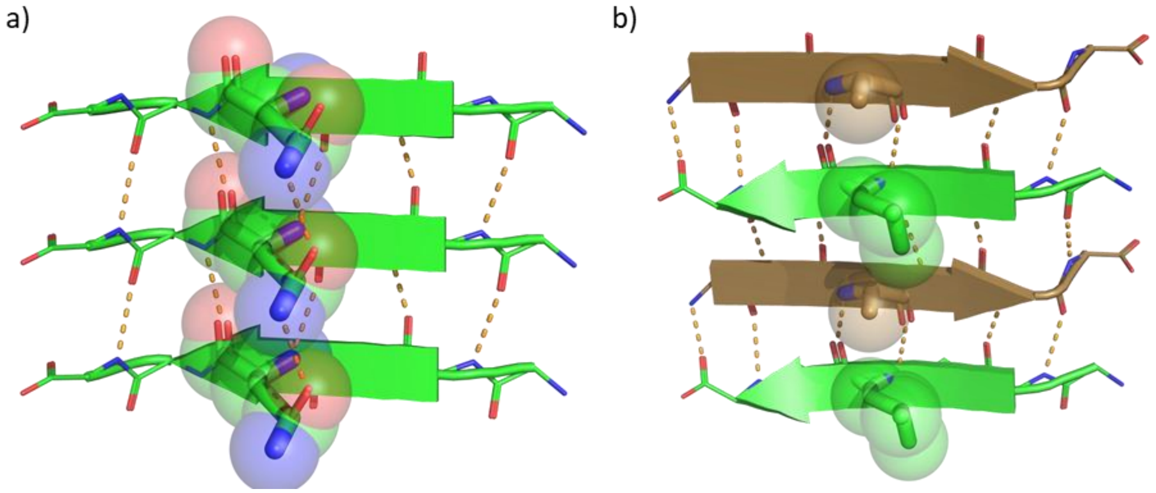

Figure S1. Examples of amino acid side chain ladders. They form stabilizing infinite side-chain/side-chain interactions (catemer synthons) on one side of a β-sheet APR structure. **a)** Gln-Gln side-chain ladder in a parallel β-sheet (PDB ID: 1YJO), **b)** Ala-Leu side-chain ladder in an antiparallel β-sheet amyloid (PDB ID: 2OMQ).

Table S4. Interacting residue pairs of the neighboring β-strands of antiparallel APRs. Side chains of such pairs can form catemer interactions within the β-sheets, resulting in ladders. A total of 177 interacting pairs were found within these APR structures.

	A	L	G	S	V	F	I	Y	T	M	E	W	K	N	H	Q	D	R	C	P
A	5	9	7	2	0	1	4	1	4	3	2	1	4	0	0	0	0	0	0	0
L	9	7	2	1	3	4	3	2	1	3	1	0	1	2	1	1	0	0	0	0
G	7	2	7	6	3	2	1	5	3	2	0	1	0	0	0	1	0	0	0	0
S	2	1	6	2	2	3	1	3	4	0	0	1	0	3	1	1	0	0	0	0
V	0	3	3	2	6	6	4	2	0	2	0	0	1	0	0	0	0	0	0	0
F	1	4	2	3	6	1	0	0	0	1	1	4	0	0	1	0	2	0	0	0
I	4	3	1	1	4	0	3	0	0	1	2	0	2	0	0	1	0	0	0	0
Y	1	2	5	3	2	0	0	0	1	1	3	1	0	0	0	0	0	0	0	0
T	4	1	3	4	0	0	0	1	0	0	0	0	0	0	0	0	0	0	0	0
M	3	3	2	0	2	1	1	1	0	1	0	0	0	0	0	0	0	0	0	0
E	2	1	0	0	0	1	2	3	0	0	0	0	0	0	0	0	0	1	0	0
W	1	0	1	1	0	4	0	1	0	0	0	0	0	0	0	0	0	0	0	0
K	4	1	0	0	1	0	2	0	0	0	0	0	0	0	0	0	0	0	0	0
N	0	2	0	3	0	0	0	0	0	0	0	0	0	0	0	0	0	0	0	0
H	0	1	0	1	0	1	0	0	0	0	0	0	0	0	1	0	0	0	0	0
Q	0	1	1	1	0	0	1	0	0	0	0	0	0	0	0	0	0	0	0	0
D	0	0	0	0	0	2	0	0	0	0	0	0	0	0	0	0	1	0	0	0
R	0	0	0	0	0	0	0	0	0	0	1	0	0	0	0	0	0	0	0	0
C	0	0	0	0	0	0	0	0	0	0	0	0	0	0	0	0	0	0	0	0
P	0	0	0	0	0	0	0	0	0	0	0	0	0	0	0	0	0	0	0	0

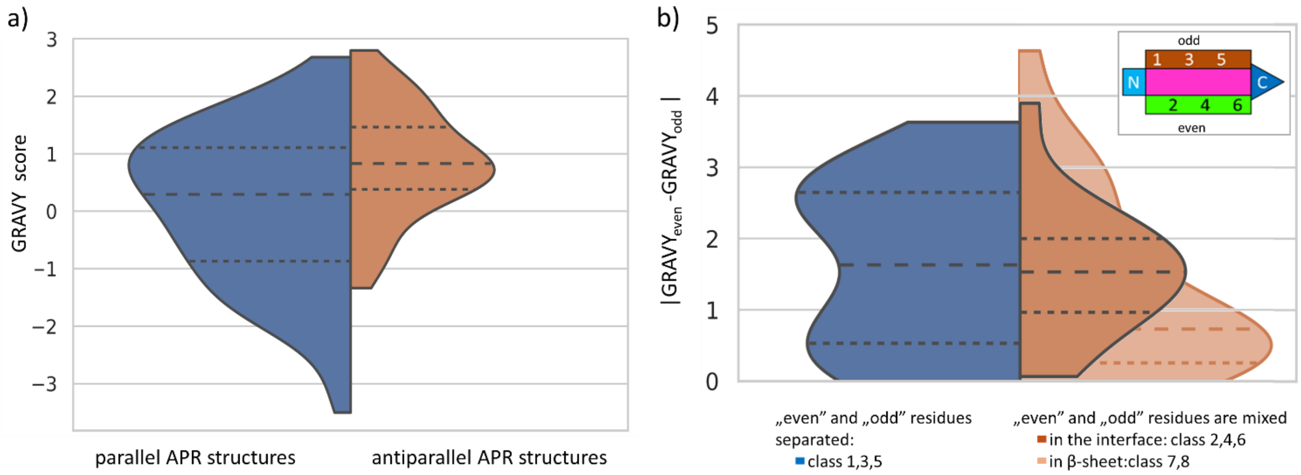

Figure S2. The distribution of the hydrophobicity (GRAVY index[1]) of APRs. a) Distribution of the GRAVY scores averaged for a polypeptide sequence within all parallel structures (blue) and all antiparallel β-sheet structures (orange) is shown as violin plots. **b)** Comparison of the hydrophobicity of the two sides of hexapeptides is shown as the absolute value of the difference of average GRAVY scores of residues on the "odd" and "even" sides of the peptide ("odd" side: 1st, 3rd, 5th residues, "even" side: 2nd, 4th and 6th residues, as shown for the β-stranded hexapeptide in the figure inset). In topological classes 1, 3 and 5, the "even" and "odd" residues are separated in different zippers (blue); while in other classes the amino acid composition of the zippers is mixed (they contain residues from both sides of the peptide) (shades of orange).

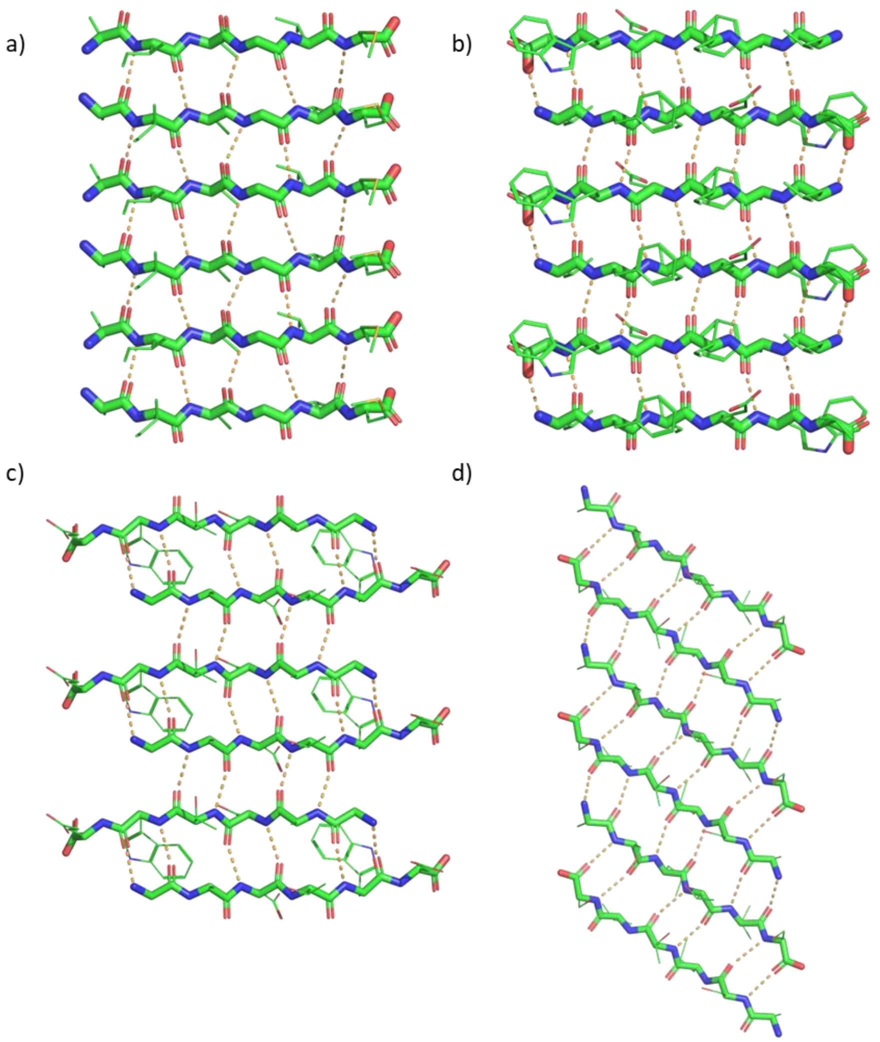

Figure S3. Comparison of the parallel and in-register and of the out-of-register antiparallel H-bond patterns. Less backbone H-bonds are formed for the out-of-register arrangement of β-strands. **a)** In register arrangement of parallel β-strands with the example of AIIGLM (PDB ID: 2Y3J, class 2). **b)** In register arrangement of antiparallel β-strands with the example of LFIEWL (PDB ID: 8ANN, class 7). **c)** Antiparallel β-sheet with overhanging ends of β-strands with the example of AADTWE (PDB ID: 6C3T, class 6). The C-terminal residue Glu is in an overhanging position. Each of the β-strands forms (6+4) backbone H-bonds. **d)** Out-of-register arrangement of β-strands, exemplified by ASLTVS (PDB ID: 6DJO, class 5-like). Each of the β-strands form (6+6) backbone H-bonds, with a vertical fibril axis. Note that the β-strands are not perpendicular to the fibril axis.

Supplementary Methods

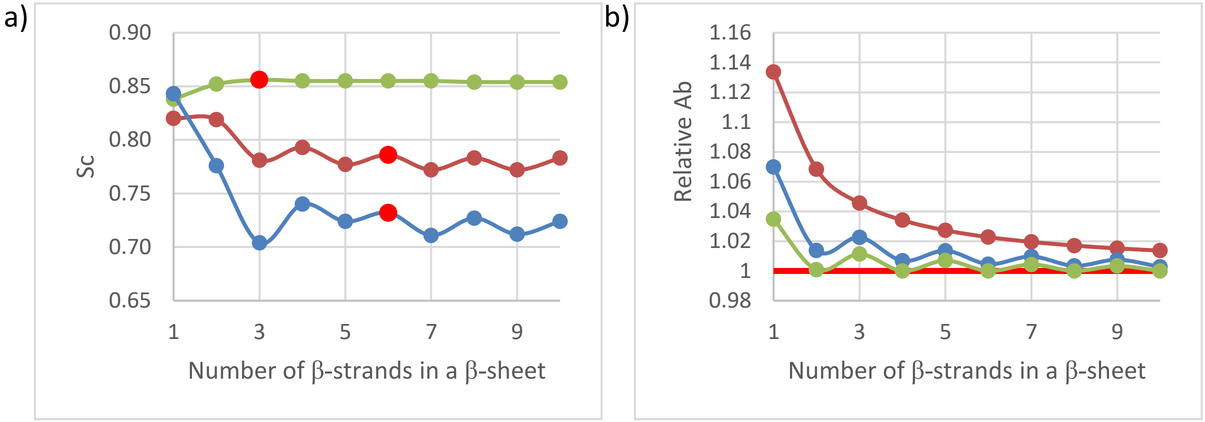

Figure S4. Dependence of calculated Sc and Ab values on system size. The descriptor values Sc **a)** and Ab values per peptide chain **b)** calculated using different numbers of β-strands within β-sheets. Three examples are shown: a parallel β-sheet with one chain as repeating unit (green, PDB ID: 1YJO; a parallel β-sheet with two chains as repeating unit (blue, PDB ID: 2Y3J); an antiparallel β-sheet (two chains as repeating unit, orange, PDB ID:2OMQ).

- **a)** Sc is the median of the distribution of the scalar products of the normal vectors of the molecular surfaces of the β-sheets, calculated in grid points. There is also a cut-off zone around the edges of the interface to minimize the effect of poor fit around the edges[2]. The relative size of the poorly fitted edges of the β-sheet pairs decreases with increasing number of β-strands and converges to a limit. When the repeating unit contains two β-strands instead of one, Sc oscillates around an average value. The change of the calculated value with increasing number of repeating units is very small beyond 3 repeating units. The red dots show the number of β-strands we recommend (Further analysis is shown in Figure S5).
- **b)** The Ab value presented here is solvent-accessible area of the two β-sheets buried[3] in the zipper divided by the number of β-strands to compensate for the system size. The first and last β-strands of a β-sheet have different contributions to the Ab values compared to the middle β-strands (for reasons similar to Sc) because they are exposed. Ab values also oscillate for structures containing two β-strands per repeating unit. These effects diminish with increasing structure size: the plot of Ab as a function of the number of β-strands converges to a limit of infinitely large structure (red line). However, the limiting value can also be calculated by considering only the area of the central β-strand(s) (i.e., the repeating unit).

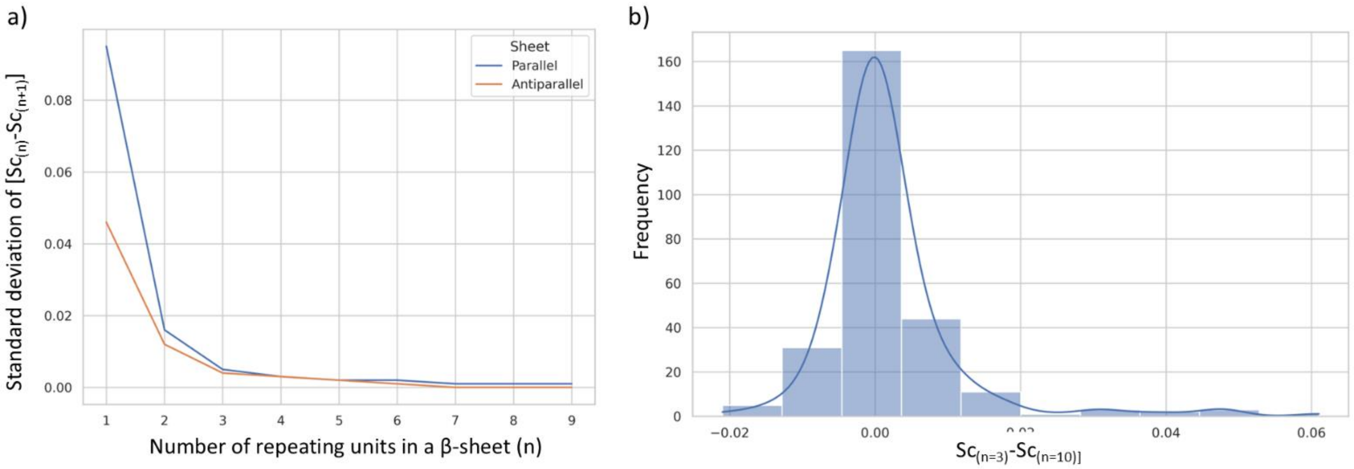

Figure S5. **Dependence of the bias of Sc (caused by the outermost peptide chain) on system size.** Sc is calculated for all 267 interfaces for the sizes of the β-sheets varying from n=1 to 10 repeating units (i.e. 1 to 10 peptide chains per sheet for parallel β-sheet structures and 2 to 20 peptide chains per sheet for the antiparallel ones). Bias of Sc is estimated by difference of Sc calculated for different system sizes.

- **a)** Standard deviation of changes of Sc values upon growing the β-sheet structures by one repeating unit [$Sc_{(n)}$-$Sc_{(n+1)}$] is diminishing with increasing the structures. For the proposed size for calculations (n=3) it is 0.005 and 0.004 for parallel and antiparallel structures.
- **b)** Distribution of the difference $Sc_{(3)}$-$Sc_{(10)}$ – that is difference of Sc for structures of 3 repeating units (the proposed definition of Sc) and 10 repeating units (as an approximation for an infinite structure). The distribution has a first quartile (Q1) of -0.002 and a third quartile (Q3) of 0.003, the average is 0.002(9). The distribution is skewed towards the right, meaning Sc is overestimated in some cases. For 8 out of the 267 interfaces, $Sc_{(3)}$-$Sc_{(10)}$ is larger than 0.030 (PDB structures: 2OL9, 4R0W, 5K2H, 5KNZ, 5WHP, 6J60, 7SXN, 8ANG). In these 8 cases there is either a solvent molecule or an unfilled cavity inside the interface. In these cases, the surface of the contacting region is small compared to the cut-off region.

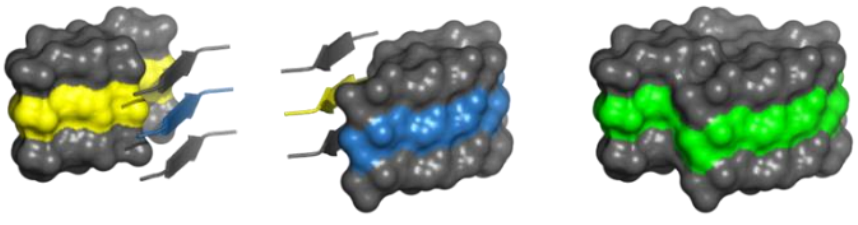

$$[\,(\,SASA_A \;+\; SASA_B) \;-\; SASA_U\;]\,/2 \;=\; Ab$$

Figure S6. Structural regions for surface calculation as part of a uniform method for calculating of Ab, Sc and SDi values for APR crystal structures. (Example shown for a parallel β-sheet structure, PDB ID: 1YJO). For the calculation of Ab, parts of the solvent accessible surfaces are calculated for the middle chains of sets of 3 repeating units: the two β-sheets separately ($SASA_A$, $SASA_B$ in yellow and blue respectively) and the two complementary β-sheets ($SASA_U$ in green).

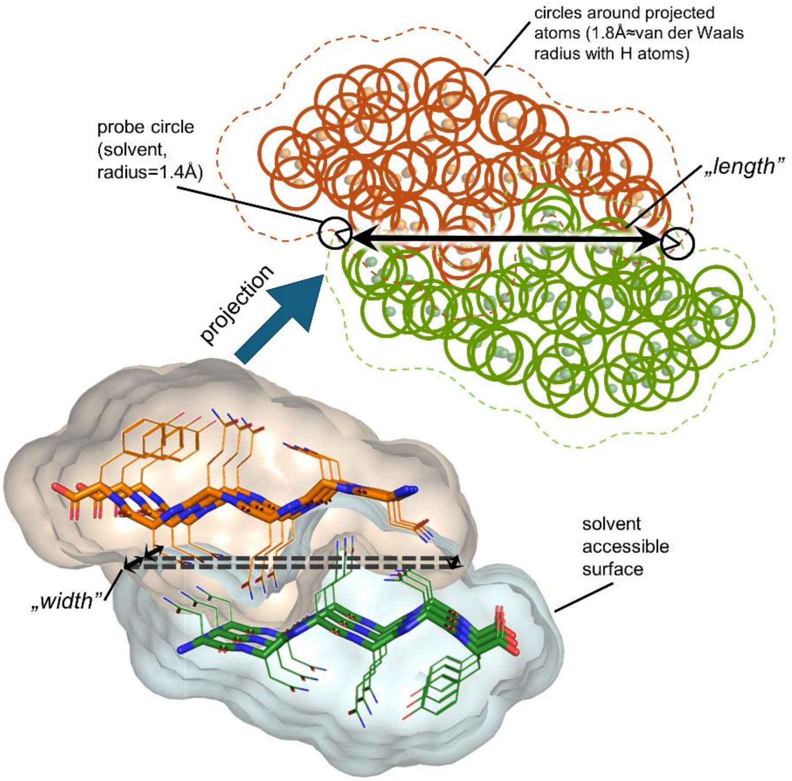

Figure S7. Definition of SDi. For the surface detail index the buried surface area (Ab, solvent-accessible surface loss of a peptide chain within the zipper region, formula is shown in Figure S5.) is divided by the area of a flat surface. The flat surface is considered to be a rectangle: The width of the rectangle is the distance between the β-strands (lower part of the figure). The length of the rectangle is calculated from the projection of the structure along the β-sheet axis (upper part of the figure) - positions of a probe circle (water molecule) are located where it touches projections of both β-sheet surfaces (similar to the calculation of the solvent-accessible surface, but in 2D; black circles). The "length" of the rectangle is the distance between the points of both circles being equal distance from the closest projected atoms of both β-sheets. Note that due to the projection method, the area of the rectangle will be an upper estimate of the area of the irregularly shaped flat surface corresponding to a structure. Note, because of the projection method, the area of the rectangle will be an upper estimation of that of the irregularly shaped surface

Additional analysis of Sc, Ab and SDi distributions

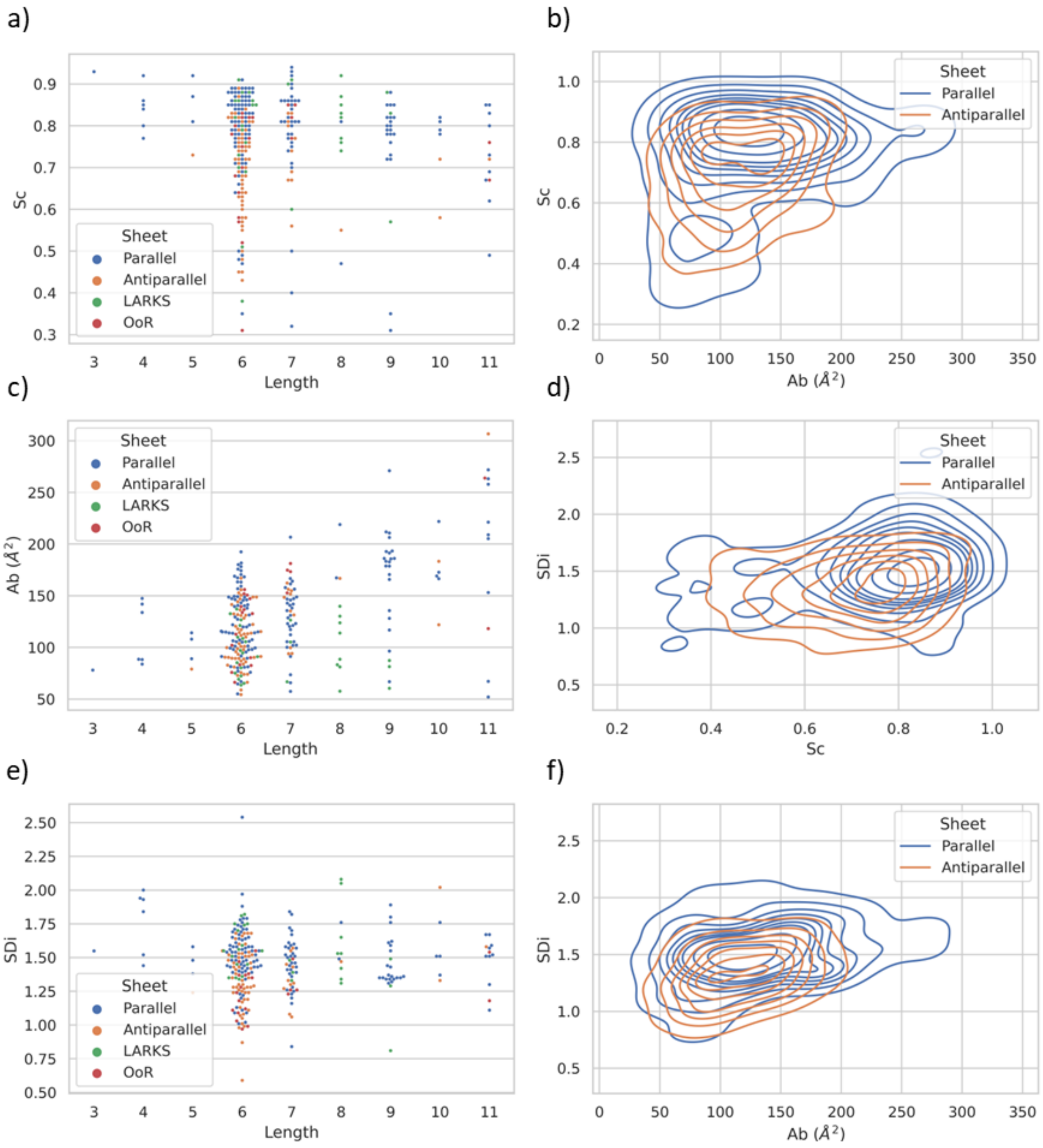

Figure S8. Ab, Sc and SDi distribution plots for different peptide lengths and their correlation. a), c) e) Distribution of descriptor values among all APR peptide structures of different oligopeptide lengths shown as swarm plots. **b), d), f)** Kernel density estimation plots of the three used descriptors (Sc, Ab and SDi) of the interfaces.

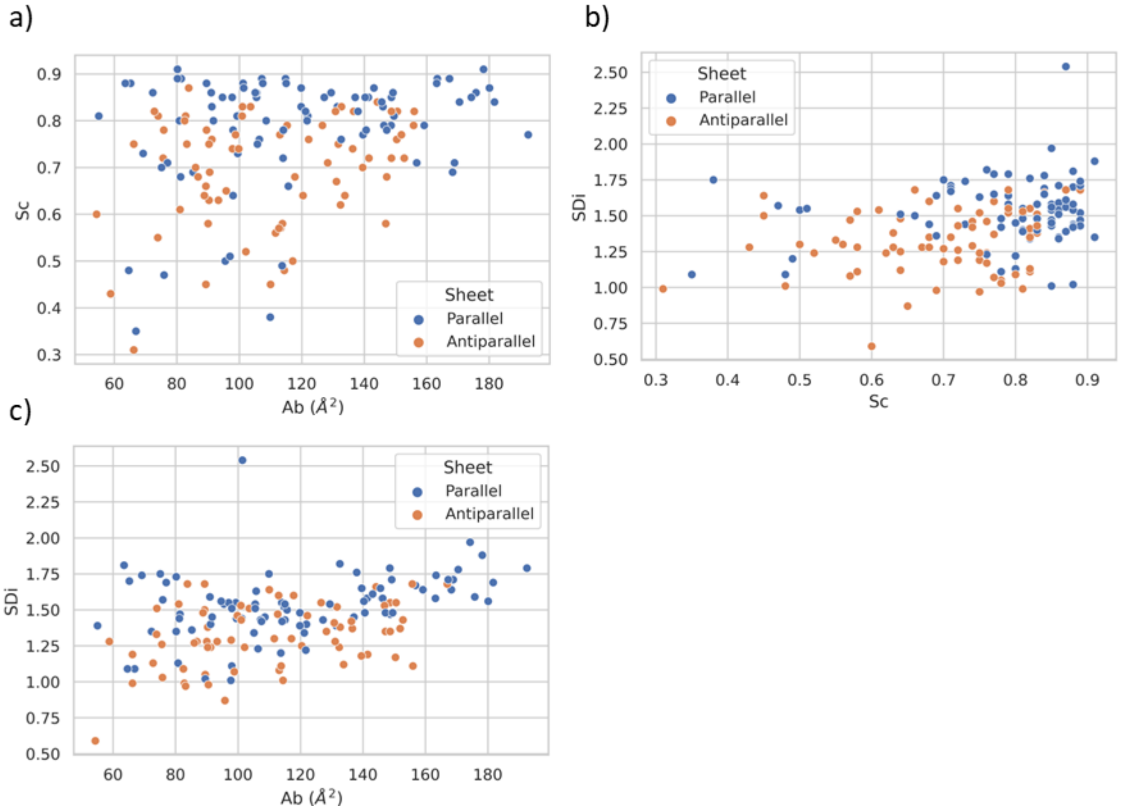

Figure S9. Co-distribution of amyloid interface descriptors in hexapeptide structures, projections. 2D projections of the 3D space of Ab, Sc and SDi values: **a)** Sc vs. Ab, **b)** SDi, vs. Sc **c)** Sc vs. Ab.

Supplementary results for APR quaternary structure

Relationship between amyloid topology and crystallographic space groups: The APR crystal structures belong to different space group symmetries. Since topological classes determine the organization of adjacent chains by means of class-characteristic twofold rotation and/or twofold screw axis symmetries, there are some space groups typical for these classes: The space group symmetry is related to the internal symmetry operators of the sandwiched β-sheet pairs[4] (i.e., the topological class). (However, these symmetries can be broken if there are more chains with different conformations in the unit cell; e.g., PDB structure 6FGR belongs to class 1, and its space group is P1). There are also some quaternary structure related preferences too: the tilted packing pattern is usually monoclinic, while the brick wall pattern is orthorhombic. The following space groups occur within the PDB structures of APRs: P1, $P2_1$, $P2_12_12_1$, $P2_12_12$, C2, I222, I2 ($P2_1$ being the most common one; Table S1).

Interestingly, in about one third of the structures there is a screw axis parallel to the z-axis that is independent of the internal class symmetry (e.g., PDB structure 2OLX belongs to class 1, and its space group is $P2_12_12_1$). The presence of this extra screw axis creates new 3D packing patterns: for example, the fishbone packing pattern can be derived from the straight packing, and a herringbone pattern from the tilted packing (Figure 7.d).

When a highly displaced interface is combined with the extra screw axis it can create small interfaces with topologies that differ from the topological class of the major interfaces within the structure. For example, 2ONW has small class 4 interfaces in a class 1 structure; 2ONA (Figure 9); and 8ANL has class 8 interfaces in class 7; class 3 interfaces in a class 1 structure 2OMM. Such combined topologies are referred to as mixed class.[5] Within the current set of APR structures there are 9 mixed class structures (referring to “main class” and “secondary class” in Table S1).

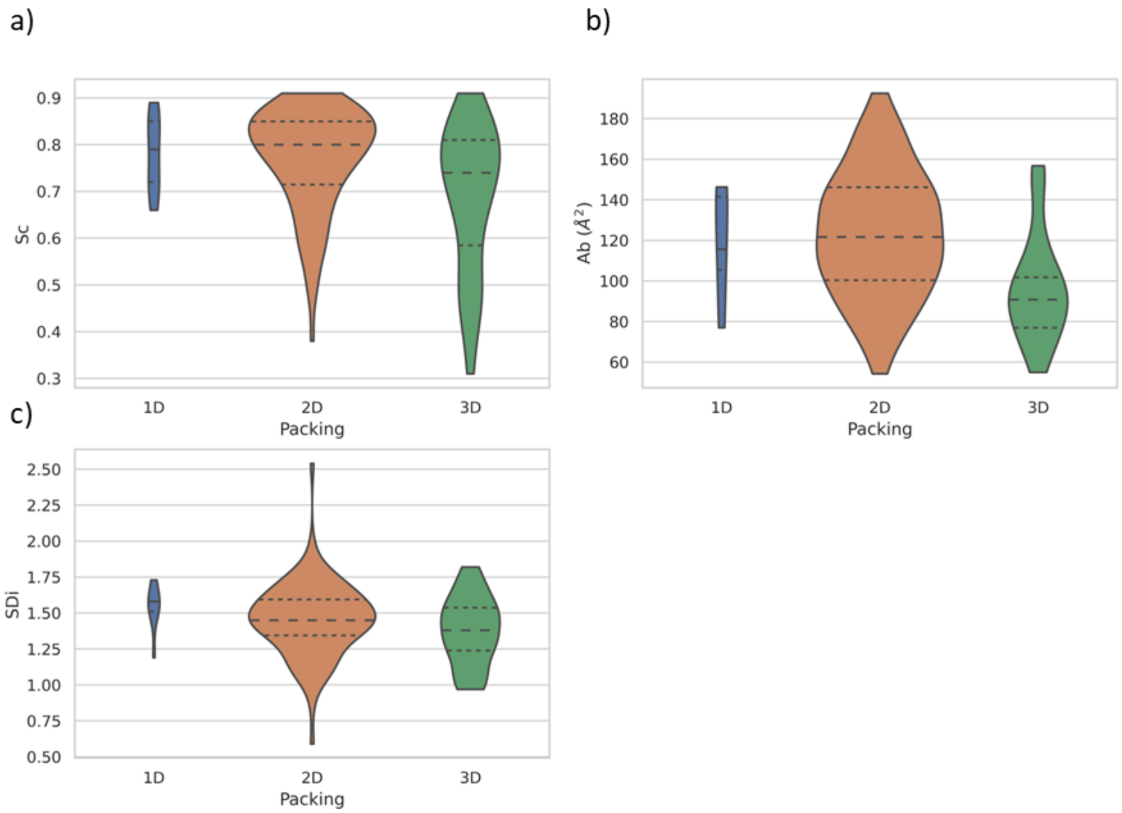

Figure S10. Distribution of descriptor values for different quaternary structure topologies. The distributions are shown for hexapeptide structures of different packing types determined by the networks of the interfaces (quaternary structure: 1D, 2D and 3D interaction networks). **a)** Sc, **b)** Ab, **c)** SDi

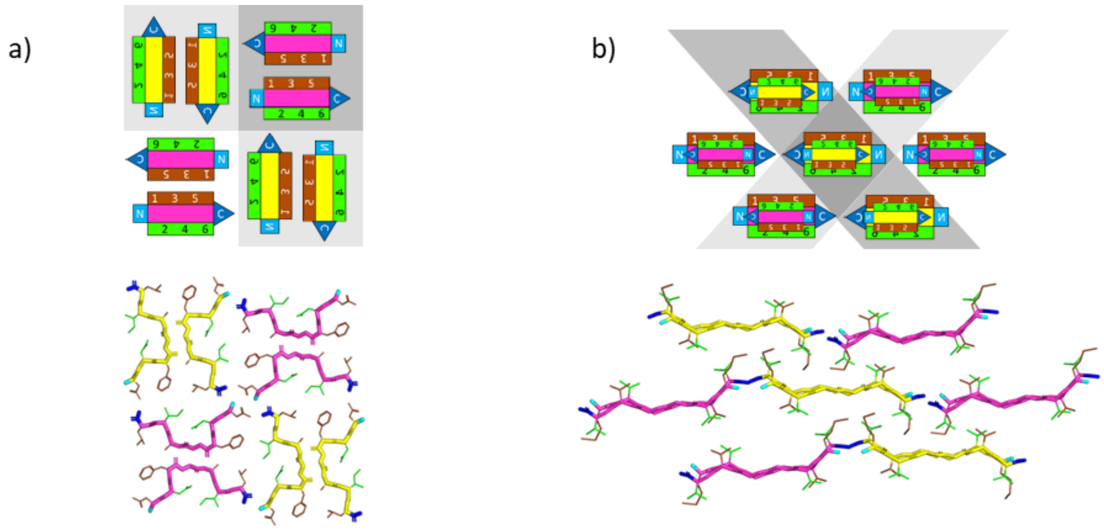

Figure S11. Atypical quaternary level structures (examples). a) Basket weave pattern of LARKS structures **b)** An orthorhombic structure with an extra screw axis compared to the majority of the structure. This pattern allows class 7 (light grey) and class 8 (dark grey) interfaces (PDB IDs: **a)** 3DGJ, **b)** 2ONA).

a) b)

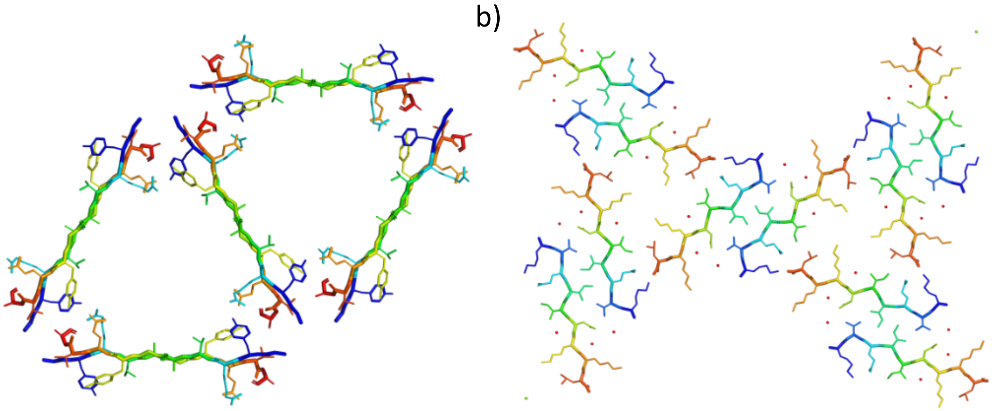

Figure S12. Trigonal or hexagonal structures of APR oligopeptides. Two examples of the unusual triangular amyloid β-sheet nanopores are shown. **a)** monolayer triangular antiparallel β-sheet, 7VI5; **b)** bilayer triangular parallel β-sheet. There are four known examples with trigonal or hexagonal space groups. In some ways they are similar to the β-helixes. These structures are outside the scope of the original topological class system because one side of the β-sheet is complementary to two other β-sheets (instead of one), and the angle between the β-sheets is 120° and the β-sheets form a trigonal prism arrangement, that encloses a threefold screw axis. A sixfold screw axis may be present at the ends of the β-sheets. Solvent channels are present around the axes. Here the standard topological categories of β-sheet arrangements (parallel/antiparallel, antifacial/equifacial) should be supplemented with others: Trigonal symmetry allows only face-to-face and up-to-up packing (classical topological classes 1, 5, 7, 10 contain the combinations of these types of arrangements). In one of the structures (6NK4) only one side of the β-sheet forms a trigonal interface, while the other forms a classical zipper region, a two-layer β-sandwich is formed around the trigonal prism-shaped nanopore. We recommend that these structures be classified with their traditional counterparts (class 1,5,7 or 10) with an additional label indicating whether they are trigonal (t) or double-layered trigonal (dt).

References

(1) Kyte, J.; Doolittle, R. F. A Simple Method for Displaying the Hydropathic Character of a Protein. *Journal of Molecular Biology* **1982**, *157* (1), 105–132. https://doi.org/10.1016/0022-2836(82)90515-0.

(2) Lawrence, M. C.; Colman, P. M. Shape Complementarity at Protein/Protein Interfaces. *Journal of Molecular Biology* **1993**, *234* (4), 946–950. https://doi.org/10.1006/jmbi.1993.1648.

(3) Lee, B.; Richards, F. M. The Interpretation of Protein Structures: Estimation of Static Accessibility. *Journal of Molecular Biology* **1971**, *55* (3), 379-IN4. https://doi.org/10.1016/0022-2836(71)90324-X.

(4) Stroud, J. C. The Zipper Groups of the Amyloid State of Proteins. *Acta Cryst D* **2013**, *69* (4), 540–545. https://doi.org/10.1107/S0907444912050548.

(5) Horváth, D.; Dürvanger, Z.; K. Menyhárd, D.; Sulyok-Eiler, M.; Bencs, F.; Gyulai, G.; Horváth, P.; Taricska, N.; Perczel, A. Polymorphic Amyloid Nanostructures of Hormone Peptides Involved in Glucose Homeostasis Display Reversible Amyloid Formation. *Nat Commun* **2023**, *14* (1), 4621. https://doi.org/10.1038/s41467-023-40294-x.
